# m6A-modified LINC02418 induces transcriptional and post-transcriptional modification of CTNNB1 via interacting with YBX1 and IGF2BP1 in colorectal cancer

**DOI:** 10.1038/s41420-025-02365-4

**Published:** 2025-03-13

**Authors:** Hao Zhang, Ye Han, Chengwei Wu, Siying Wang, Mingquan Chen, Qian Xu, Hong Wei, Xianli Zhou, Guiyu Wang

**Affiliations:** 1 Department of Colorectal Surgery, the Second Affiliated Hospital of Harbin Medical University, Harbin, 150000 China; 2 In-Patient Ultrasound Department, the Second Affiliated Hospital of Harbin Medical University, Harbin, 150000 China

**Keywords:** Cell biology, Cancer

## Abstract

Colorectal cancer (CRC) represents a significant menace to human health, but its molecular pathogenesis remains unclear. Herein, we explored the functional role of LINC02418 in CRC progression. The function of LINC02418 in CRC was determined through vitro and in vivo experiments. The molecular mechanism of LINC02418 in CRC was explored by quantitative real-time PCR (qPCR) analyses, western blot, luciferase reporter assay, methylated RNA immunoprecipitation (MeRIP) assay, RNA pull-down, RNA immunoprecipitation (RIP) assay and chromatin immunoprecipitation (ChIP) assay. The results revealed that LINC02418 expression was upregulated in CRC tissues and the high expression of LINC02418 was related to unfavorable survival of CRC patients. Besides, knockdown of LINC02418 expression resulted in the inhibition of proliferation and metastasis of CRC cells in vitro and in vivo. Mechanistically, we found METTL3-mediated m6A modification induced the aberrant expression of LINC02418 in CRC. LINC02418 could interact with YBX1 and enhance YBX1 DNA-binding ability to the CTNNB1 promoter, resulting in transcriptional activation of CTNNB1. In the post-transcriptional stage, LINC02418 could also enhance CTNNB1 stability by promoting the interaction between IGF2BP1 protein and CTNNB1 mRNA. What is more, LINC02418 expression could be transcriptionally enhanced by YBX1 protein. Collectively, this study unveils a novel oncogenic mechanism for LINC02418 in CRC and the LINC02418 might be a novel therapeutic target in CRC treatment.

## Introduction

Colorectal cancer (CRC) ranks among the most widespread and deadly cancers on a global scale. Each year witnesses more than 1.8 million fresh instances being diagnosed across various regions with a notable surge observed amongst younger cohorts (< 50 years old), especially within developed countries [1]. Multiple risk factors contribute to this escalated incidence encompassing hereditary APC and MLH1 gene mutations alongside obesity, unhealthy dietary patterns, and sedentary lifestyles [2]. Disturbingly enough, nearly 60% of recently identified CRC cases manifest at an advanced stage, thereby amplifying mortality rates significantly [3]. Despite noteworthy progressions witnessed lately concerning CRC treatment modalities, patient prognosis continues to be unfavorable [4–7]. Therefore, gaining a thorough understanding of the fundamental molecular mechanisms that drive the progressive and aggressive characteristics observed in CRC cells will undeniably play a crucial role in developing personalized therapeutic strategies based on biomarkers for each individual with CRC.

Genome-wide analyses and functional studies have revealed a wide range of functional mechanisms within long noncoding RNAs (lncRNAs), which are deemed as RNA molecules longer than 200 nucleotides without protein-coding ability but can produce noncoding transcripts [8, 9]. By participating in tumor signaling pathways and targeting specific genes through interactions with DNA, RNA and proteins, lncRNAs can modulate transcriptional processes, posttranscriptional events, translation mechanisms, and epigenetic modifications to influence apoptosis, cellular proliferation, DNA damage repair pathways, cell cycle regulation among other biological processes [10–14]. Despite the increasing annotation of lncRNAs in recent decades, additional elucidation of the functions and potential regulatory pathways of unrecognized lncRNAs in CRC is essential for the investigation of therapeutic targets and potential diagnostic markers.

Our research team has analyzed lncRNAs in the GEO databases (GSE102340, GSE83889 and GSE81558), with LINC02418 demonstrating higher expression in colorectal cancer tissues compared to normal mucosa. LINC02418 is a lncRNA located on chromosome 12q24.33, with no coding potentiality. Recently, although there are some explorations for LINC02418 in colorectal cancer [15–17], all previous studies only focused on the ceRNA mechanism and the intricate mechanism of LINC02418’s participation in colorectal cancer is still not thoroughly understood. In this research, we have determined LINC02418 is elevated in colorectal cancer and knockdown LINC02418 significantly diminishes the proliferation and metastasis of CRC cells both in vitro and in vivo. We found that the upregulation of LINC02418 is attributed to N6-methyladenosine (m6A) modification mediated by methyltransferase like 3 (METTL3), which could result in the stabilization of LINC02418. Besides, we have revealed that LINC02418 plays a significant part in the activation of Wnt pathway and promoting colorectal cancer progression via binding to YBX1 and IGF2BP1 protein to perform transcriptional and post-transcriptional regulation of CTNNB1. Furthermore, YBX1 can bind to the promoter region of LINC02418 and induce LINC02418 transcriptional upregulation.

## Results

### LINC02418 expression is elevated in colorectal cancer and correlates with poor clinical outcomes

**Fig. 1:**
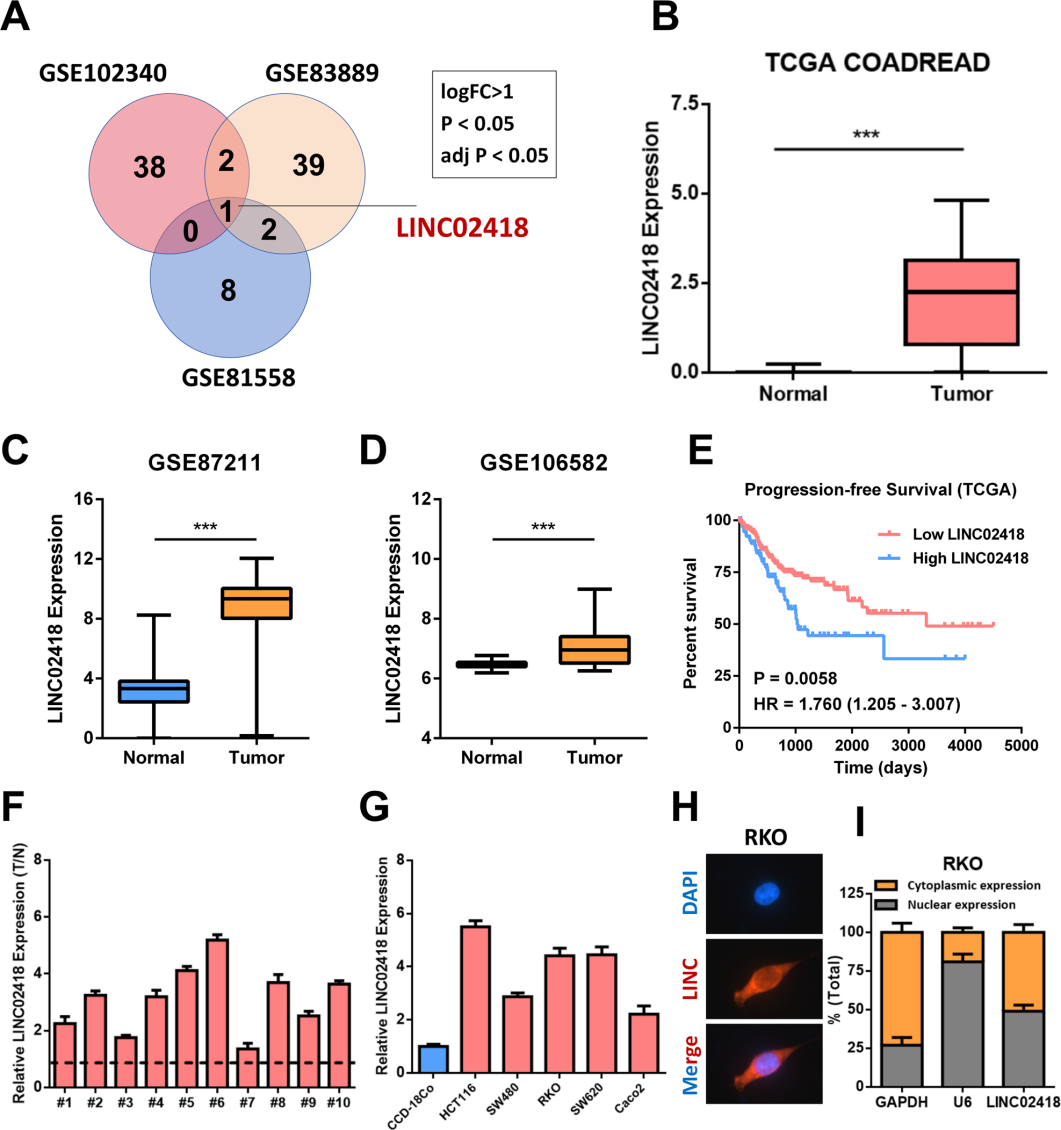
LINC02418 is overexpressed in colorectal carcinoma and correlates with poor clinical outcomes. **A** Venn diagram of highly expressed lncRNAs in CRC tissues of the three GEO datasets (GSE102340, GSE83889 and GSE81558) revealed the cancer-promoting lncRNA, LINC02418. (**B**) Expression of LINC02418 in CRC and non-tumor tissues in the TCGA dataset. **C**–**D** Expression of LINC02418 in CRC and nontumor tissues in the GSE87211 and GSE106582 datasets. **E** High LINC02418 levels predict worse PFS based on the TCGA dataset. **F** The expression levels of LINC02418 were assessed in 10 pairs of clinical colorectal cancer (CRC) samples and their corresponding adjacent tissues using qPCR assay. **G** A qPCR analysis was employed to quantify the protein expression levels of LINC02418 in human colorectal cancer (CRC) cell lines, as well as the normal colon cell line CCD-18Co. **H** The subcellular localization of LINC02418 (red) in RKO was measured by RNA FISH (Scale bar, 30μm). **I** Quantification of LINC02418 distribution percentage was measured by nucleocytoplasmic fractionation.

To begin with, we analyzed the highly expressed lncRNAs (logFC > 1, *p* < 0.05 and adj *p* < 0.05) in CRC tissues utilizing the GSE102340, GSE83889 and GSE81558 databases, and the Venn diagram indicated a significant upregulation of LINC02418 in CRC tissues compared to normal tissues (Fig. 1A), which was also evidenced by analyses of TCGA COADREAD, GSE87211 and GSE106582 databases (Fig. 1B–D). Furthermore, Kaplan-Meier analysis using TCGA data revealed high LINC02418 expression were related to unfavorable progression-free survival outcomes (*P* = 0.0058) (Fig. 1E). To further determine the importance of LINC02418 in CRC, we performed qPCR on 10 pairs of tissue samples, confirming a significant increase in LINC02418 expression in CRC tissues (Fig. 1F). Additionally, elevated levels of LINC02418 expression were observed in colorectal cancer cells (HCT116, SW480, RKO, SW620 and Caco2) when compared with the CCD-18Co (Fig. 1G). We also conducted subcellular localization analysis of LINC02418 in CRC cell and found that LINC02418 was localized both in the nucleus and cytoplasm (Fig. 1H-I). Overall, our findings support the conclusion that LINC02418 is significantly overexpressed and positively correlated with poor survival in colorectal cancer.

### LINC02418 knockdown inhibits proliferation and metastasis of colorectal cancer cells in vitro and vivo

**Fig. 2:**
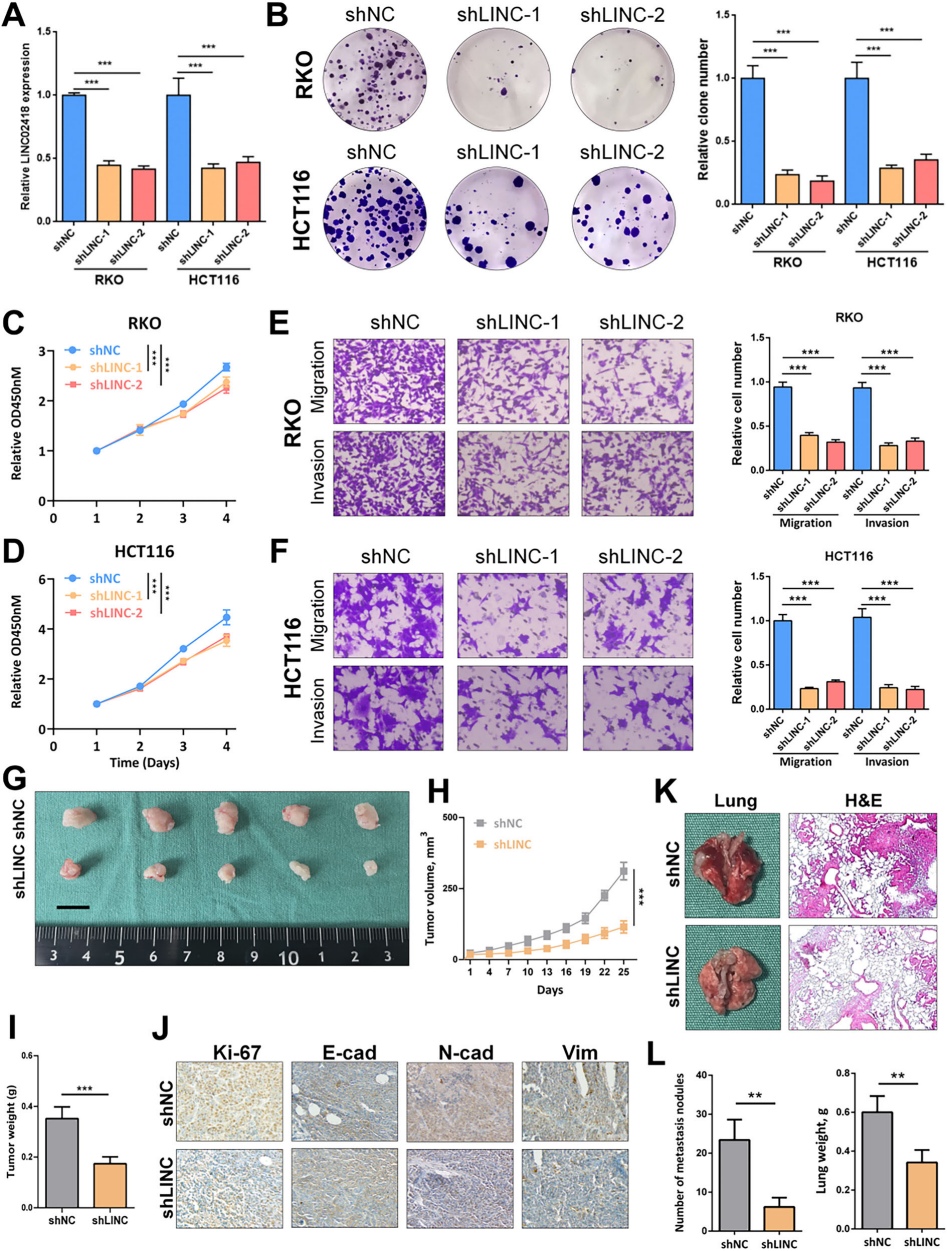
LINC02418 knockdown inhibits proliferation and metastasis of colorectal cancer cells in vitro and vivo. **A** The expression of LINC02418 was determined by qPCR in RKO and HCT116 cells with LINC02418 knockdown. **B** Representative images and quantification of colony formation assay depicting the growth of RKO and HCT116 cells under LINC02418 knockdown. **C, D** CCK8 assay indicates the change in cell viability of RKO and HCT116 cells under LINC0248 knockdown. **E**–**F** Representative images and quantification of transwell assay depicting the migration and invasion abilities of RKO and HCT116 cells with LINC02418 knockdown. **G**–**H** Tumor volumes in the shNC and shLINC0248 groups were measured every 3 days and growth curves were plotted (*n* = 5 per group). **I** Tumor weights of shNC and shLINC02418 groups in the xenograft model. **J** Representative IHC staining images of Ki67, E-cadherin, N-cadherin and Vimentin for indicated tumor tissues. (**K**) Representative lung images and H&E staining analysis of mouse lung sections showing tumor lesions (*n* = 5 per group). **L** The number of metastatic nodules in the lungs was counted (*n* = 5 per group). Lung weight was counted (*n* = 5 per group).

To explore the functional roles of LINC02418, we conducted LINC02418 knockdown using two specific short hairpin RNAs (sh1 and sh2) in RKO and HCT116 cells (Fig. 2A). Colony formation assay and CCK-8 assay revealed that LINC02418 knockdown contributed to a decrease in cell growth rate and reduced colony formation ability in both RKO and HCT116 cells (Fig. 2B–D). Additionally, the transwell assay demonstrated that LINC02418 knockdown also led to reduced cell migration and invasion abilities (Fig. 2E–F). Besides, we successfully induced overexpression of LINC02418 in RKO and Caco2 cells (Fig. S1A-B), which induced enhanced cell proliferation capability and colony formation potential (Fig. S1C-H). Furthermore, our findings from the transwell assay demonstrated overexpression of LINC02418 notably increased the migration and invasion abilities of RKO and Caco2 cells (Fig. S1I–L).

To investigate the impact of LINC02418 on colorectal cancer (CRC) cell proliferation in vivo, a subcutaneous xenograft experiment was conducted using HCT116 cells with and without LINC02418 knockdown. This experimental approach aimed to evaluate the influence of LINC02418 on CRC growth. The tumor volumes were meticulously monitored during tumor progression, and subsequently, the subcutaneous tumors were harvested for further analysis. Remarkably, both the volume and weight of xenograft tumors in the shLINC02418 group showed notable delays compared to the shNC group (Fig. 2G–I). IHC analysis exhibited a notable reduction in Ki-67 expression as well as EMT-related markers within the shLINC02418 group (Fig. 2J). To investigate the impact of LINC02418 on colorectal cancer (CRC) metastasis in vivo, the lung metastasis model was established. HCT116 cells from two groups (shNC and shLINC02418) were intravenously injected into nude mice via the tail vein. The histopathological features of the lung tissues were determined by HE staining **(**Fig. 2K). Moreover, the shLINC02418 group exhibited a significant reduction in both the number of metastatic tumor nodules and lung weight, compared to the shNC group (Fig. 2L). Collectively, our results strongly suggested that knockdown of LINC02418 suppress both CRC cell proliferation and metastasis in vitro and in vivo.

### m6 A modification was involved in the upregulation of LINC02418 in CRC

**Fig. 3:**
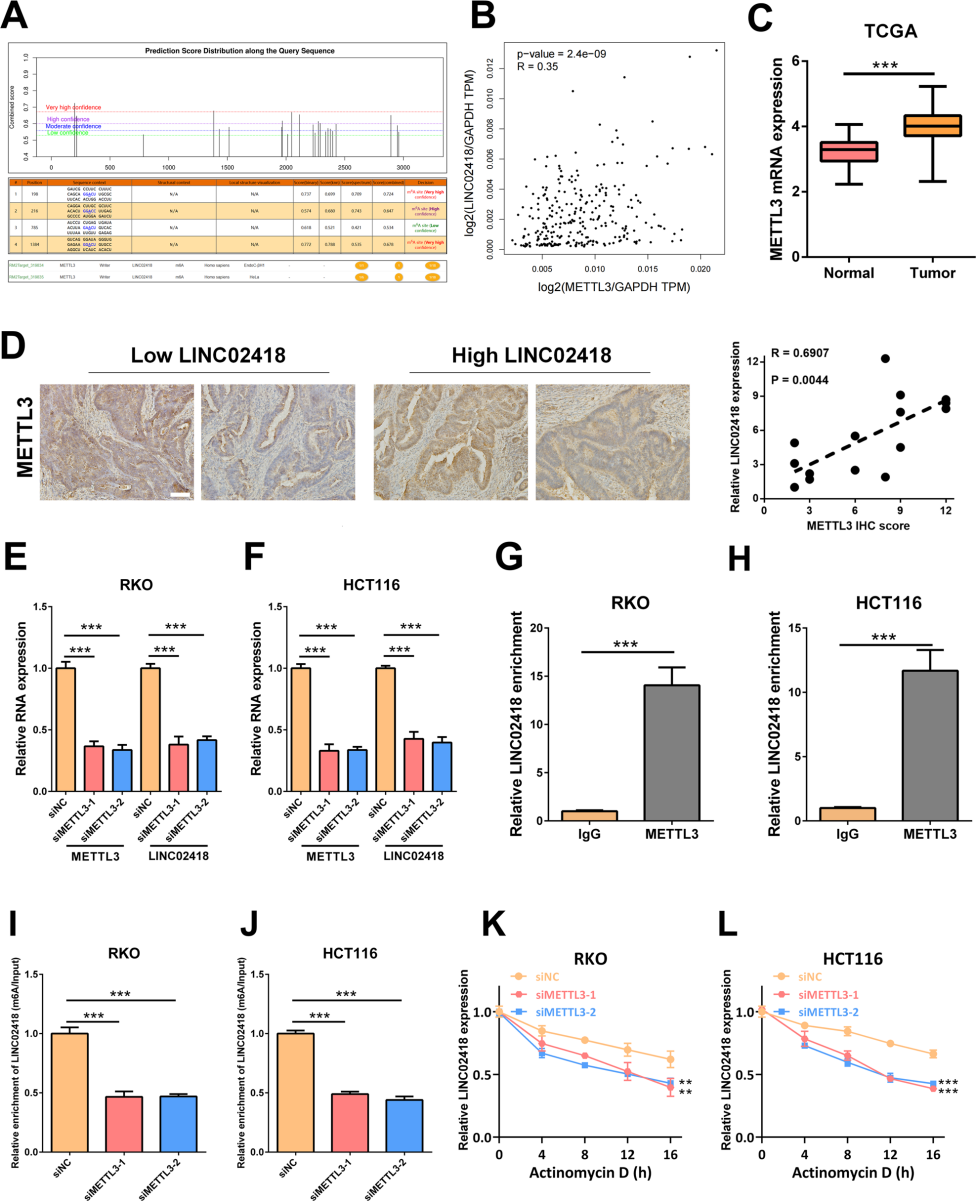
m6 A modification is involved in the upregulation of LINC02418 in CRC. **A** The potential m6A modification sites of LINC02418 were predicted by SRAMP and the m6A methylation-related “writer” METTL3 was predicted through the RM2Target. **B** The correlation between the LINC02418 and METTL3 expression levels in the TCGA database. **C** Expression of METTL3 in CRC and non-tumor tissues in the TCGA dataset. **D** Positive correlation between LINC02418 and METTL3 expression levels in pathological samples (Scale bar, 400 μm). **E–F** LINC02418 expression was detected by qPCR after METTL3 knockdown in RKO and HCT116 cells. **G**–**H** RIP assay showed the content of LINC02418 immunoprecipitated by METTL3. **I–J** METTL3 induced m6A modification of LINC02418 in RKO and HCT116 cells. **K–L** The RNA stability of LINC02418 in RKO and HCT116 cells with and without METTL3 knockdown.

The underlying mechanism responsible for the upregulation of LINC02418 expression remains elusive. Recent research has indicated that N6-methyladenosine (m6A) modification plays a role in the stabilization and expression of lncRNAs in cancer. The SRAMP predictive analysis revealed a high abundance of m6A modification sites in LINC02418 [18], suggesting that LINC02418 is likely to undergo m6A methylation and the m6A methylation-related “writer” METTL3 was predicted through the RM2Target website [19] (Fig. 3A). Notably, the expression of METTL3 was positively correlated with LINC02418 expression and significantly upregulated in CRC tissues (Fig. 3B-C). Moreover, we used pathological samples (*n* = 15) to further validate the correlation between LINC02418 and METTL3 expression. The results showed that the pathological samples from the high LINC02418 expression group exhibited significantly higher METTL3 expression levels, compared to low LINC02418 expression group (stratified by median LINC02418 expression level). Furthermore, Pearson correlation analysis demonstrated a strong positive correlation between LINC02418 and METTL3 expression levels (*R* = 0.6907, *P* = 0.0044) (Fig. 3D). Subsequently, we explored the effect of METTL3 on LINC02418 expression, and the results revealed that the expression of LINC02418 was significantly reduced in RKO and HCT116 cells with METTL3 knockdown (Fig. 3E-F). Research reports indicated that m6A modification is also regulated by other m6A writers including METTL14 and WTAP [20, 21]. Therefore, to further underscore the specificity and importance of METTL3-dependent m6A modification in the regulation of LINC02418 stability and expression, we also assessed the LINC02418 level after knocking down METTL14 or WTAP in RKO and HCT116 cells. However, the results suggested that neither METTL14 nor WTAP affected LINC02418 expression (Fig. S2A-D). The RIP assay showed that there was an interaction between METTL3 and LINC02418 in CRC cell (Fig. 3G-H). More importantly, the results of MeRIP-qPCR showed that the m6A modification of LINC02418 was notably decreased in RKO and HCT116 cells with METTL3 knockdown (Fig. 3I-J). In addition, we explored the effect of METTL3 on the stability of LINC02418 and found that the stability of LINC02418 was decreased in CRC cells with METTL3 knockdown **(**Fig. 3K-L**)**. To further determine the clinical significance of LINC02418 m6A modification, we also performed a survival analysis for CRC patients with different LINC02418 m6A modification level. The results suggested that patients in the high LINC02418 m6A modification group had a worse overall-survival (*P* = 0.0328, HR = 1.760, 95%CI = 1.125–12.410) (Fig. S2E).

### LINC02418 plays a positive role in the regulation of the Wnt pathway in CRC

**Fig. 4:**
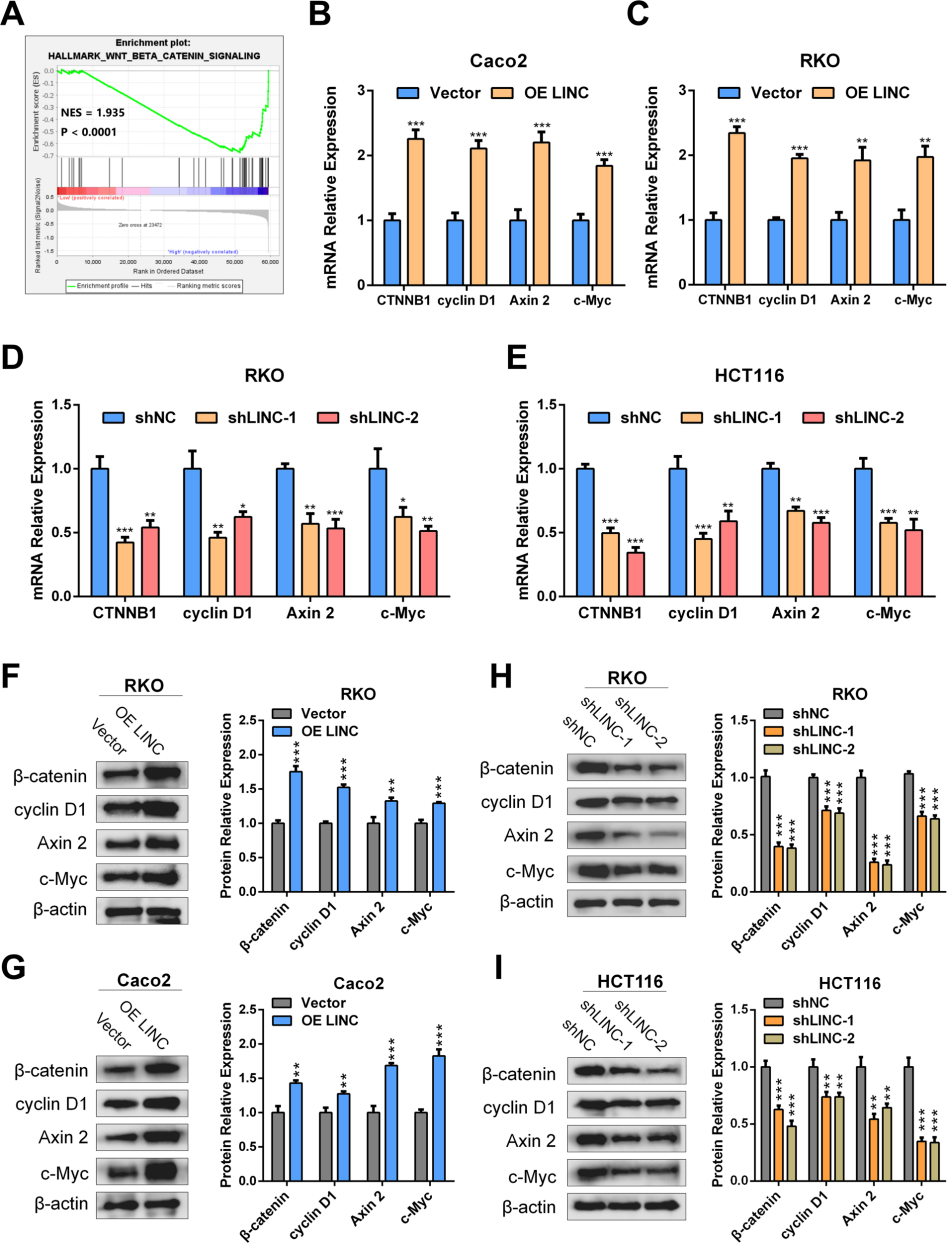
LINC02418 plays a positive role in the regulation of the Wnt/β-catenin signaling pathway in CRC. **A** GSEA analysis suggested that there is a positive correlation between LINC02418 and the Wnt signaling pathway in TCGA COADREAD dataset. **B–C** mRNA level of β-catenin and typical Wnt signaling pathway-targeted genes were detected by qPCR after LINC02418 overexpression in RKO and Caco2 cells. **D–E** mRNA level of β-catenin and typical Wnt signaling pathway-targeted genes were detected by qPCR after LINC02418 overexpression in RKO and HCT116 cells. **F–G** Protein level of β-catenin and typical Wnt signaling pathway-targeted genes were detected by western blot after LINC02418 overexpression in RKO and Caco2 cells. **H–I** Protein level of β-catenin and typical Wnt signaling pathway-targeted genes were detected by western blot after LINC02418 knockdown in RKO and HCT116 cells.

To explore the underlying mechanism by which LINC02418 promotes CRC progression, we performed Gene Set Enrichment Analysis (GSEA) using the RNA-seq data from the TCGA COADREAD dataset and found a positive correlation between LINC02418 and the Wnt signaling pathway (Figs. 4A and S3A). Besides, the expression level of LINC02418 was positively correlated with β-catenin and Wnt signaling pathway-targeted genes (c-Myc, Axin2, and cyclin D1) in TCGA and GEO databases (GSE106582, GSE87211 and GSE83889) (Fig. S3B). Subsequently, qPCR analysis and WB assay were performed to validate our findings, assessing the mRNA and protein expression levels of β-catenin, cyclin D1, Axin2, and c-Myc following LINC02418 overexpression and knockdown. Our findings suggested that in comparison to the negative control group, there was a significant increase in mRNA levels of β-catenin and Wnt/β-catenin signaling pathway-targeted genes in CRC cells overexpressing LINC02418 (Fig. 4B-C). Conversely, knockdown of LINC02418 resulted in decreased mRNA levels of Wnt/β-catenin signaling pathway-related genes (Fig. 4D-E). Furthermore, western blot analysis revealed similar results for β-catenin, cyclin D1, Axin2 and c-Myc protein levels following both LINC02418 overexpression and knockdown (Fig. 4F-I).

### LINC02418 promotes transcription of CTNNB1 by interacting with YBX1

**Fig. 5:**
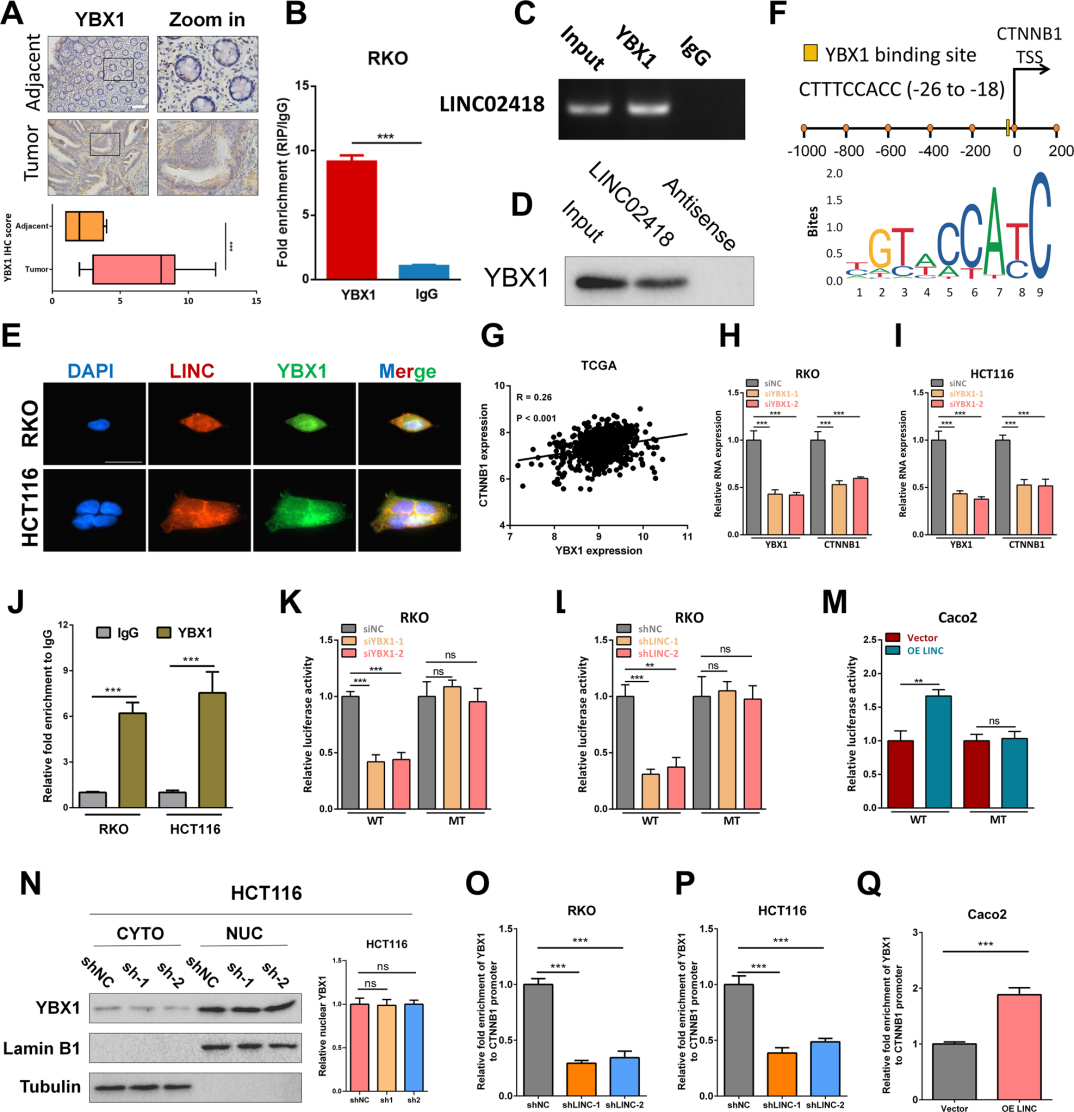
LINC02418 induces transcription of CTNNB1 by interacting with YBX1. **A** Representative images of IHC staining in 16 pairs of CRC and adjacent tissues for YBX1 (Scale bar, 400 μm). **B**–**C** Relative LINC02418 enrichment levels after RIP in RKO cell. **D** Western blot for YBX1 after RNA pull-down in RKO cell. **E** The co-localization of LINC02418 and YBX1 protein in CRC cells (Scale bar, 30μm). **F** Analysis using the JASPAR database show the predicted YBX1 binding motifs in the β-catenin promoter. **G** The correlation between the mRNA levels of β-catenin and YBX1 in the TCGA database. **H**–**I** β-catenin expression was detected by q-PCR after YBX1 knockdown in RKO and HCT116 cells. **J** YBX1 enrichment at the β-catenin promoter was measured using a YBX1 antibody to perform the ChIP assay. (**K**) The dual-luciferase analysis for β-catenin transcriptional activity was performed in RKO cell with or without YXB1 knockdown. **L** The dual-luciferase analysis for β-catenin transcriptional activity was performed in RKO cells with or without LINC02418 knockdown. **M** The dual-luciferase analysis for β-catenin transcriptional activity was performed in Caco2 cell with or without LINC02418 overexpression. **N** Western blot analysis to show the YBX1 expression in the nucleus and cytoplasm of HCT116 cell with LINC02418 knockdown. **O**–**P** CHIP-qPCR showed the binding ability of YBX1 to the CTNNB1 promoter could be inhibited in RKO and HCT116 cells with LINC02418 knockdown. **Q** CHIP-qPCR showed the binding ability of YBX1 to the CTNNB1 promoter could be induced in Caco2 cell with LINC02418 overexpression.

Earlier results had demonstrated the significant regulatory role of LINC02418 in modulating the expression level of β-catenin (translated by CTNNB1), a pivotal gene associated with Wnt pathway. LncRNAs typically exert their influence on the expression of downstream gene mRNAs through the competitive endogenous RNA mechanism. However, analysis using the starBase database did not reveal any overlapping microRNAs between LINC02418 and CTNNB1. LINC02418 may potentially modulate the expression of CTNNB1 through alternative mechanisms. Therefore, we aimed to identify potential protein interactors of LINC02418 with the catRAPID and RBPDB online databases [22, 23], and the results suggested LINC02418 might interact with YBX1, IGF2BP1 and YTHDC1 **(**Figure S4A**)**, which could act as a transcription factor or m6A reader, respectively. To begin with, we put the view at YBX1. The immunohistochemistry (IHC) results demonstrated that YBX1 exhibited more intense staining in colorectal cancer (CRC) tissues compared to adjacent tissues (16 normal vs. 16 tumor, *P* < 0.001). The interaction between YBX1 and LINC02418 was further assessed by RIP, and it confirmed the interaction between LINC02418 and YBX1 in CRC cells **(**Fig. 5B-C**)**. Besides, we found that in RKO cell, YBX1 was notably detectable by WB after the RNA pull-down of LINC02418, but not antisense-LINC02418 **(**Fig. 5D**)**. Immunofluorescence analysis also revealed the co-localization of LINC02418 and YBX1 in HCT116 and RKO cells **(**Fig. 5E**)**. The JASPAR analysis unveiled the presence of YBX1 binding motifs within the promoter region of CTNNB1. The site located at −26 to −18 (CTTTCCACC) upstream of the transcription start site of β-catenin was identified as the predicted binding site **(**Fig. 5F**)**. In TCGA database, the mRNA level of β-catenin was positively correlated with YBX1 **(**Fig. 5G**)**. In addition, YBX1 knockdown in CRC cells resulted in a significant decrease in β-catenin mRNA level **(**Fig. 5H-I**)**, whilst YBX1 overexpression could lead to an increase in β-catenin mRNA level **(**Figure. S4B-C**)**. Furthermore, a ChIP assay was conducted, obviously demonstrating the binding between YBX1 and the promoter region of β-catenin **(**Fig. 5J**)**. To confirm whether YBX1 could activate β-catenin transcription in CRC cells, a functional validation was carried out using a dual luciferase reporter assay, which revealed that knockdown of YBX1 significantly reduced the luciferase activity of the β-catenin-promoter-WT reporter in RKO and HCT116 cells **(**Fig. 5K and Figure. S3D**)**, while overexpression of YBX1 notably enhanced luciferase activity of the β-catenin-promoter-WT reporter in CRC cells **(**Figure. S4E-F**)**. However, no significant effect on the luciferase activity of MT β-catenin reporter was observed. Therefore, it can be concluded that YBX1 acts as a transcriptional activator for β-catenin in CRC cells.

What is more, the dual-luciferase analysis demonstrated a significant decrease regarding transcriptional activity of CTNNB1 promoter containing YBX1-binding site following knockdown of LINC02418 in CRC cells, while LINC02418 overexpression resulted in the opposite effect **(**Fig. 5L-M and Figure S5A-B**)**. However, it was noted that LINC02418 did not impact the activity of the CTNNB1 promoter containing mutant YBX1 binding sites **(**Fig. 5L-M and Figure S5A-B**)**. Subsequently, our investigation aimed to elucidate the underlying mechanisms through which LINC02418 modulated the transcriptional regulatory activity of YBX1. To begin with, we found that knockdown LIN02418 did not change YBX1 protein expression significantly, as evidenced by western blot analyses **(**Fig. S5C**)**. Next, we aimed to explore the impact of LINC02418 on the subcellular distribution of YBX1, but the result indicated that there was no notable difference regarding YBX1 nuclear translocation in CRC cells after knocking down and overexpressing LINC02418 **(**Fig. 5N and Figure S5D**)**. Therefore, we examined whether LINC02418 could affect YBX1 transcriptional regulatory activity by enhancing its DNA-binding activity. Interestingly, the CHIP-qPCR revealed that knockdown and overexpression of LIN02418 could inhibit and induce the CTNNB1 promoter-binding activity of YBX1, respectively **(**Figs. 5O-Q and S5E**)**. Collectively, these results provided compelling evidence supporting the positive regulatory role of LINC02418 in modulating β-catenin transcriptional activity via interacting with YBX1 and enhancing DNA-binding ability of YBX1.

### LINC02418 increases CTNNB1 stability by interacting with IGF2BP1

**Fig. 6:**
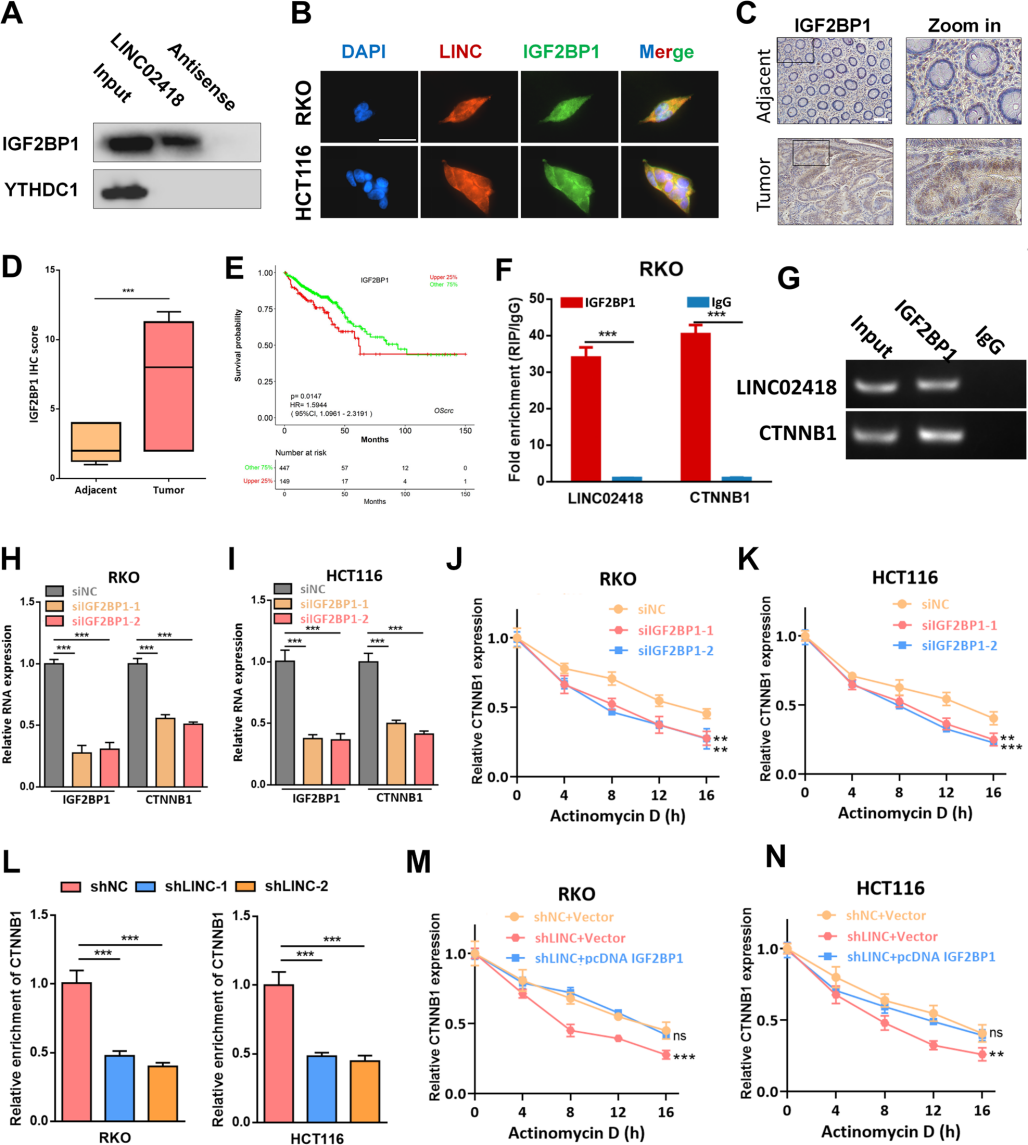
LINC02418 increases CTNNB1 stability by interacting with IGF2BP1. **A** Western blot for IGF2BP1 and YTHDC1 after RNA pull-down in RKO cell. **B** The co-localization of LINC02418 and IGF2BP1 protein in CRC cells (Scale bar, 30μm). **C–D** Representative images of IHC staining in 16 pairs of CRC and adjacent tissues for IGF2BP1 (Scale bar, 400 μm). **E** High IGF2BP1 levels predict worse OS in TCGA. **F–G** RIP assay confirmed the enrichment of IGF2BP1 with LINC02418 and CTNNB1. **H–I** CTNNB1 expression was detected by qPCR after IGF2BP1 knockdown in RKO and HCT116 cells. **J–K** The RNA stability of CTNNB1 in RKO and HCT116 cells with and without IGF2BP1 knockdown. **L** The interaction between IGF2BP1 protein and CTNNB1 mRNA was strikingly weakened by LINC02418 deficiency in RKO and HCT116 cells. **M–N** shLINC02418-mediated reduction in CTNNB1 mRNA stability could be counteracted by IGF2BP1 overexpression.

Subsequently, we analyzed the role of m6A reader in the regulation of CTNNB1 by LINC02418. We found that IGF2BP1, but not YTHDC1, was detectable after the RNA pull-down of LINC02418 **(**Fig. 6A**)**. The interaction between LINC02418 and IGF2BP1 was also confirmed by confocal images **(**Fig. 6B**)**. Besides, our findings indicate a high expression of IGF2BP1 in tumor tissues (16 normal vs. 16 tumor, *P* < 0.001) and its association with poorer overall survival in TCGA CRC patients (*P* = 0.00147) **(**Fig. 6C-E**)**. More interestingly, analysis from the starBase database suggested a potential interaction between IGF2BP1 and both LINC02418 and CTNNB1, which held significant implications as IGF2BP1 has been implicated in tumor progression through its binding to m6A modification sites for regulating target mRNA stability [24]. Notably, The products pulled down by the IGF2BP1 antibody showed a significant enrichment of CTNNB1 and LINC02418 when tested with qRT-PCR **(**Fig. 6F-G**)**. Further investigation revealed that knocking down IGF2BP1 in RKO and HCT116 cells significantly downregulated the mRNA level of CTNNB1 **(**Fig. 6H-I**)** and decreased the stability of CTNNB1 mRNA after exposure to actinomycin D **(**Fig. 6J-K**)**. What is more, we observed that the interaction between IGF2BP1 protein and CTNNB1 mRNA was notably weakened by LINC02418 deficiency in RKO and HCT116 cells **(**Fig. 6L**)**. And the shLINC02418-mediated reduction in CTNNB1 mRNA stability could be counteracted by overexpression of IGF2BP1 **(**Fig. 6M-N**)**. Moreover, it was found that the expression of IGF2BP1 remained unchanged after knocking down LINC02418, indicating that LINC02418 inhibition reduced CTNNB1 stability by decreasing the affinity of IGF2BP1 to CTNNB1 mRNA without affecting its expression level **(**Figure S5F**)**. Therefore, our findings suggested that LINC02418 could enhance CTNNB1 stability in CRC cells by promoting the interaction between IGF2BP11 protein and CTNNB1 mRNA.

### CTNNB1 overexpression reversed the effects of LINC02418 knockdown in CRC cells

**Fig. 7:**
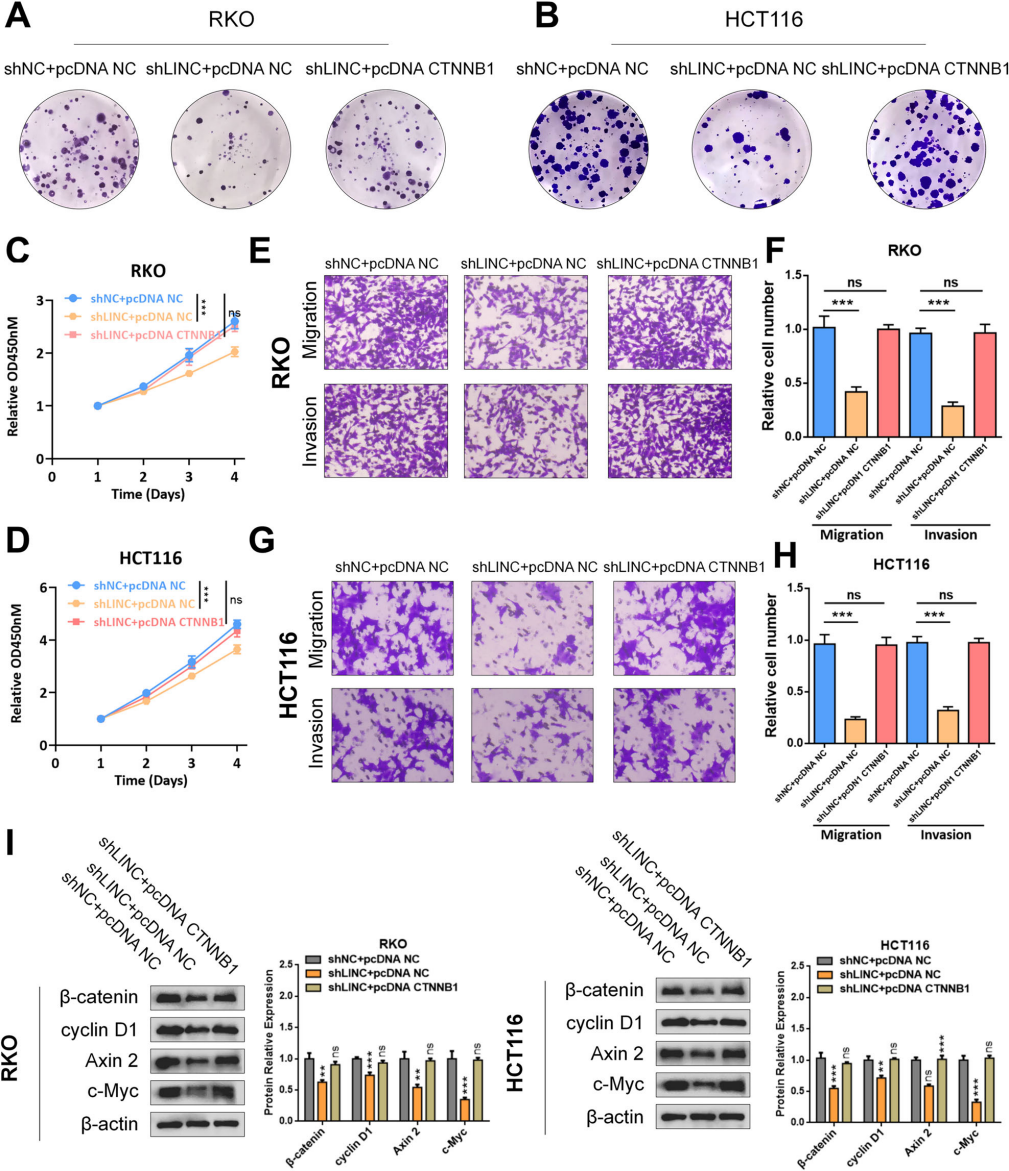
CTNNB1 overexpression reversed the effects of LINC02418 knockdown in CRC cells. **A**-**D** Colony formation and CCK8 assays indicated the change in cell proliferation ability of RKO and HCT116 cells induced by shLINC02418 was reversed by CTNNB1 overexpression. **E–H** Transwell assay indicated the change in cell migration and invasion abilities of RKO and HCT116 cells induced by shLINC02418 was reversed by CTNNB1 overexpression. **I** The protein changes of typical Wnt signaling pathway-targeted genes in RKO and HCT116 cells induced by shLINC02418 were reversed by CTNNB1 overexpression.

To further investigate the potential biological function of LINC02418 through the upregulated CTNNB1, rescue experiments were performed by transfecting CRC cells with pcDNA NC or pcDNA CTNNB1 following knockdown of LINC02418. Colony formation, CCK8 and transwell assays revealed that CTNNB1 overexpression effectively counteracted the inhibition of cell proliferation and migration induced by LINC02418 knockdown in RKO and HCT116 cells **(**Fig. 7A-H and Fig. S6**)**. Additionally, qPCR and western blotting analyses revealed that CTNNB1 overexpression reversed the decrease in expression levels of Wnt signaling pathway-targeted genes (cyclin D1, Axin2, and c-Myc) caused by LINC02418 knockdown. These findings suggest that LINC02418 exerts its biological function through CTNNB1 **(**Fig. 6I**)**.

### YBX1 could bind to the LINC02418 promoter and induce LINC02418 transcription

**Fig. 8:**
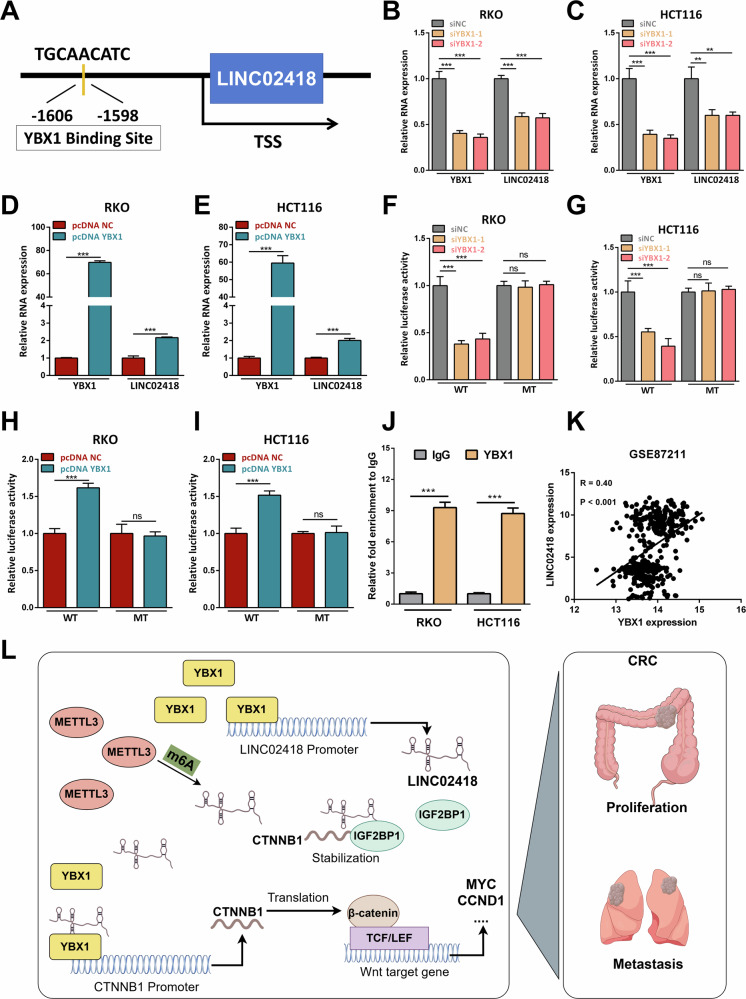
YBX1 could bind to the LINC02418 promoter and induce LINC02418 transcription. **A** The JASPAR analysis revealed potential binding of YBX1 to the promoter region of LINC02418. **B–C** LINC02418 expression was detected by qPCR after YBX1 knockdown in RKO and HCT116 cells. **D–E** LINC02418 expression was detected by qPCR after YBX1 overexpression in RKO and HCT116 cells. **F–G** The dual-luciferase analysis for LINC02418 transcriptional activity was performed in HCT116 and RKO cells with or without YBX1 knockdown. **H–I** The dual-luciferase analysis for LINC02418 transcriptional activity was performed in HCT116 and RKO cells with or without YBX1 overexpression. **J** YBX1 enrichment at the LINC02418 promoter was measured using a YBX1 antibody to perform the ChIP assay. **K** The correlation between the LINC02418 and YBX1 expression levels in the GSE87211 database. **L** Schematic representation of a model for the molecular mechanisms for LINC02418 which promotes the proliferation and metastasis of colorectal cancer.

More interestingly, the JASPAR analysis revealed potential binding of YBX1 to the promoter region of LINC02418 (location: -1606 to -1598, TGCAACATC) **(**Fig. 8A**)**. Consistently, our findings demonstrated that knockdown of YBX1 resulted in a significant decrease in LINC02418 expression level **(**Fig. 8B-C**)**, while overexpression of YBX1 led to an increase in LINC02418 expression **(**Fig. 8D-E**)**. Furthermore, luciferase reporter assays using wild and mutated LINC02418 promoters indicated that YBX1 knockdown and overexpression corresponded with reduced and increased luciferase activity in CRC cells carrying the WT reporter construct, respectively **(**Fig. 8F-I**)**. Additionally, ChIP assays conducted in HCT116 and RKO cells confirmed significant binding of YBX1 to the LINC02418 promoter **(**Fig. 8J**)**. As expected, the results from GEO database (GSE106582, GSE87211 and GSE83889) showed the expression level of LINC02418 was positively correlated with YBX1 **(**Figs. 8K and S7**)**. Therefore, our data provided evidence for direct binding of YBX1 to the LINC02418 promoter and its role in promoting LINC02418 transcription.

## Discussion

Globally, colorectal cancer (CRC) presents a significant threat to human health due to its high incidence and mortality rate. A comprehensive understanding of oncogenic genes is essential for gaining insights into molecular profiling in CRC proliferation and metastasis, as well as for developing more potent combination-based therapies. Herein, we found LINC02418 was upregulated in colorectal cancer. However, the precise molecular function and underlying mechanism of LINC02418 in promoting CRC progression remains elusive. Therefore, our study aimed to elucidate the specific function of LINC02418 and its underlying mechanism in driving the progression of colorectal cancer.

Based on the results obtained from clinical samples, our study determined that LINC02418 expression is upregulated in colorectal cancer (CRC) tissues compared to peritumoral tissues. Through a series of knockdown and overexpression experiments conducted in vitro, we have revealed a positive regulatory role for LINC02418 in CRC cell proliferation, migration, and invasion. Furthermore, we employed subcutaneous tumor models and lung metastasis models to elucidate the impact of LINC02418 on CRC proliferation and metastasis. Consistent with our vitro findings, LINC02418-knockdown significantly impairs CRC cell proliferation and metastasis in vivo. Collectively, our study highlights the pivotal and positive contribution of LINC02418 to CRC progression.

The m6A modification, a reversible epigenetic regulatory mechanism, has been extensively documented to play a crucial role in the stabilization and expression of RNAs, including lncRNAs, in the progression of cancer [25]. The dysregulated expression of numerous lncRNAs has been associated with the onset of various malignancies, and the epigenetic profiles of m6A-related lncRNAs are increasingly being acknowledged as potential prognostic biomarkers, disease stage predictors, overall survival indicators, and determinants of drug resistance in cancer [26–28]. METTL3, as a member of the RNA methyltransferase family, plays a crucial role in modulating RNA stability, degradation, and translation via m6A modification. In this study, the bioinformatics analysis successfully identified high-confidence m6A sites within the sequence of LINC02418. Subsequently, we found that the upregulation of LINC02418 is attributed to m6A modification facilitated by METTL3, which could lead to the stabilization of LINC02418.

The Wnt pathway is a highly conserved signaling cascade involved in diverse biological processes, including development, cell differentiation, proliferation, stem cell dynamics, and tissue patterning [29–32]. β-catenin, a pivotal component of the classical Wnt/β‑catenin pathway, frequently exhibits dysregulated expression in cancer [33–35]. β-catenin could translocate to the nucleus and collaborate with TCF/LEF transcription factors to activate genes that may contribute to cancer progression [36, 37]. A thorough comprehension of the functions of Wnt signaling in cellular processes and cancer progression may provide valuable perspectives on potential therapeutic targets and strategies. In this study, our GSEA analysis revealed a significant positive correlation between LINC02418 and Wnt signaling pathway. Furthermore, we observed a positive association between the expression level of LINC02418 and the β-catenin as well as typical Wnt signaling pathway-targeted genes (cyclin D1, Axin2, and c-Myc). As a potential downstream target of LINC02418, the change of β-catenin at the transcriptional level caught our attention, which is a key part for Wnt pathway and the relationship between LINC02418 and β-catenin has not been reported previously.

The Y Box Binding Protein 1, also known as YBX1 and encoded by the YBX1 gene, is a well-studied member of the highly conserved Cold Shock Protein (CSP) family [38]. As a multifunctional oncoprotein, overexpression of YBX1 has been frequently observed and is often correlated with unfavorable prognoses in human cancers [39–42]. Furthermore, YBX1 serves as a critical transcription factor and RNA-binding protein (RBP), playing a pivotal role in promoting the transcription of target genes and regulating mRNA stability and translation [43–45]. In this study, utilizing a bioinformatics approach, we predicted the interaction between LINC02418 and YBX1, which was subsequently validated using RNA pull-down and RIP assays. In this paper, we found YBX1 could bind to the promoter of β-catenin and act as a transcriptional activator for β-catenin. And we have determined LINC02418 could exert its cancer-promoting effects by interacting with YBX1 and enhancing YBX1 DNA-binding ability to the CTNNB1 promoter, which could induce the transcription of β-catenin and result in the activation of Wnt pathway. These findings offer evidence to substantiate the critical role of LINC02418/YBX1 binding in the regulation of β-catenin transcription and Wnt signaling pathway activation. What is more, we found YBX1 was a transcription factor for LINC02418 based on bioinformatic analyses and determined the binding site of YBX1 in the LINC02418 promoter with the JASPAR tool (−1606 to −1598). Besides, our ChIP-qPCR and dual luciferase reporter assays provided compelling evidence that YBX1 could functionally bind to the LINC02418 promoter and increase the transcription of LINC02418.

IGF2BP1 also plays an important role in the activation of the Wnt pathway caused by LINC02418. The insulin-like growth factor-2 mRNA-binding protein 1 (IGF2BP1), a member of a conserved family of single-stranded RNA-binding proteins (IGF2BP1-3) [46, 47]. In numerous in vivo and in vitro studies, a variety of cancer-related mRNAs have been identified, including PTEN, ACTB, MAPK4, MKI67, c-MYC, and CD44. IGF2BP1 has been recognized for its regulatory role in these mRNAs, playing crucial functions in the proliferation and growth of both normal and tumor tissues, as well as in tumor cell adhesion, apoptosis, migration, and invasion [24, 48]. In this study, we first discovered that IGF2BP1 formed a complex with LINC02418 and CTNNB1. Moreover, LINC02418 enhanced CTNNB1 stability in CRC cells by promoting the interaction between IGF2BP1 protein and CTNNB1 mRNA. This discovery provided a fresh perspective on the mechanistic role of LINC02418 in cancer.

Although there are some explorations for LINC02418 in CRC [15–17], all previous studies on LINC02418 in CRC have focused on the ceRNA mechanism and our research still uncovers several novel insights. Firstly, we are the first to determine that LINC02418 activates the Wnt signaling pathway, a critical pathway in tumor progression, which highlights a previously unrecognized role of LINC02418 in CRC. Secondly, we demonstrate that LINC02418 interacts with RNA-binding proteins (RBPs) YBX1 and IGF2BP1, regulating CTNNB1 expression through transcriptional and post-transcriptional processes, moving beyond the traditional ceRNA-based mechanism. Additionally, we identify two key upstream regulatory mechanisms: METTL3 enhances LINC02418 stability via m6A modification, explaining its overexpression in CRC, and YBX1 acts both as an RBP and a transcription factor, directly promoting LINC02418 transcription. Therefore, our findings provide a comprehensive understanding of the regulatory network involving LINC02418 in CRC, expanding its known functions and offering new directions for future research.

In this study, LINC02418 shows significant potential as both a diagnostic and prognostic biomarker for colorectal cancer (CRC). Its high expression in CRC tissues, compared to normal tissues, makes it an attractive candidate for molecular diagnostic panels, especially when combined with other CRC biomarkers. Detecting elevated LINC02418 in blood or tumor samples might aid early detection and improve patient outcomes. Besides, its association with worse progression-free survival also suggests it could be used to monitor disease progression and help personalize treatment plans. Additionally, LINC02418’s oncogenic function in CRC is driven by its interactions with proteins like YBX1 and IGF2BP1, positioning LINC02418 as a promising therapeutic target. Approaches like oligonucleotide-based therapeutics, including antisense oligonucleotides and RNA interference (RNAi)-mediated gene silencing [49, 50], can be employed to inhibit the expression of LINC02418 for therapeutic applications. Besides, high-throughput screening techniques could be used to identify small molecule inhibitors to block the interaction between LINC02418 and its binding partners, YBX1 and IGF2BP1, offering another avenue for targeted therapy and further supporting the therapeutic potential of targeting LINC02418. Combining these strategies could provide a multifaceted approach to CRC treatment, enhancing therapeutic efficacy and targeting critical pathways involved in tumor progression.

In conclusion, our study determined that LINC02418 expression is upregulated in colorectal cancer. Additionally, we found that LINC02418 knockdown significantly inhibited the proliferation, migration, and invasion in colorectal cancer cells. Mechanistically, we found that the upregulation of LINC02418 is attributed to m6A modification mediated by METTL3, which could result in the stabilization of LINC02418. Besides, our results revealed LINC02418 could interact with YBX1 and enhance YBX1 DNA-binding ability to the CTNNB1 promoter, leading to the upregulation of β-catenin and activation of Wnt pathway. LINC02418 could also enhance CTNNB1 stability by promoting the interaction between IGF2BP1 protein and CTNNB1 mRNA. Moreover, we discovered that YBX1 directly binds to the promoter region of LINC02418 and facilitates LINC02418 transcription **(**Fig. 8L**)**. Therefore, targeting LINC02418 may represent a promising therapeutic strategy in treating CRC patients.

## Materials and methods

### Clinical specimens

Human CRC tissues and adjacent non-malignant tissues were procured from the Second Affiliated Hospital of Harbin Medical University. None of the patients had received radiotherapy or chemotherapy before surgery. The histopathological confirmation of CRC or non-malignant tissue status was performed by two independent pathologists to ensure accuracy and reliability. All specimens obtained informed consent from patients and were approved by the hospital’s ethics committee (YJSDW2022-134).

### Cell lines

CRC cell lines were sourced from the Cell Bank of the Type Culture Collection Committee, Chinese Academy of Sciences (Shanghai, China). All cells were cultured in a medium supplemented with 10% fetal bovine serum (FBS, GIBCO, Carlsbad, CA, USA) under a controlled environment of 5% CO_2_ at 37 °C.

### Cell transfection

The lentiviral vectors, including lenti-shLINC02418, lenti-LINC02418 (containing full-length LINC02418), and their respective control vectors, were custom-designed and obtained from GeneChem (Shanghai, China). Cells that successfully underwent transfection were subjected to a 2-week selection period with puromycin (Sigma-Aldrich Corp., St. Louis, MO, USA). The specific sequences of shLINC-1 and shLINC-2 can be found in Supplemental Table 1. Lipofectamine 2000 reagent was employed following the manufacturer’s protocol in the transfection of siRNAs and pcDNA. The specific sequences of siRNAs can be found in Supplemental Table 2.

### Immunohistochemical (IHC) staining and scoring

The tissue samples were initially treated with dewaxing and hydrating, followed by microwave restoration. To prevent non-specific antigens from interfering, the endogenous peroxidase blocker H_2_O_2_ was introduced. Subsequently, the samples were incubated overnight at 4 °C with primary antibodies, and then exposed to the appropriate secondary antibody. After adding DAB for visualization purposes, hematoxylin was utilized to stain the cell nucleus. The immunohistochemistry staining of the colorectal cancer (CRC) tissue microarray was independently evaluated by three observers, including at least one pathologist. Intensity scoring involved categorizing into four levels: negative (0), weak (1), moderate (2), and strong (3). Similarly, positive cell percentage scoring ranged from 0–25% (1), 26–50% (2), 51–75% (3), to 76–100% (4). Finally, each tissue’s IHC score was determined by multiplying the intensity score with the percentage of positive cells.

### RNA extraction and quantitative PCR

Total RNA for CRC cell line was isolated using TRIzol reagent (#15596026, Invitrogen). Subsequently, 2 μg RNA was reverse-transcribed utilizing the High-Capacity cDNA Reverse Transcription Kit (#FSQ101, TOYOBO) following the protocol. DNA concentration was determined with the Nanodrop 2000 spectrophotometer. For qPCR, 2 μg of cDNA was utilized in combination with SYBR Green reagent (#A25742, Invitrogen) on the Applied Biosystem 7500 qPCR system. The relative RNA expression was measured by the 2^−ΔΔCt^ method and normalized to GAPDH. Supplementary Table 3 provides detailed information regarding primer sequences used in this study.

### Western blotting assay and antibodies

The Western blotting procedure was conducted using established methods as previously described. Total protein for CRC cell line was obtained and quantified by the BCA Protein Assay Kit (#P1513, APPLYGEN) following the manufacturer’s instructions. For Western blotting analysis, proteins were separated on a 10% sodium dodecyl sulfate-polyacrylamide gel electrophoresis (SDS-PAGE) and subsequently transferred onto a nitrocellulose membrane. To prevent nonspecific binding, the membrane was blocked for 1.5 h at room temperature by 5% non-fat milk in Tris-buffered saline. Primary antibodies using were then incubated overnight at 4 °C followed by incubation with secondary antibodies for 1.5 h at room temperature. The signal was visualized with the ECL Kit (#P1010, APPLYGEN). Then, the image was measured utilizing the Bio-Rad ChemiDoc MP system. Please refer to Supplemental Table 4 for detailed information regarding the antibodies used in this study.

### Cell proliferation and colony formation assays

Cell proliferation was assessed by the Cell Counting Kit-8 (CCK8) (#C0038, Beyotime) cell proliferation assays. CRC cells (1000/per well) were plated in 96-well plates and incubated for different time points: 24 h, 48 h, 72 h and 96 h. Then, the cells were incubated with CCK-8 solution for an additional 2 hours to evaluate cell proliferation with the absorbance 450 nm.

For the colony formation assay, 500 CRC cells were seeded into dishes with 12-well plates and cultured for two weeks. Colonies were fixed using a solution containing paraformaldehyde (4%) and stained with crystal violet solution (2%). Subsequently, colonies were observed under a microscope, photographed, and their numbers were recorded.

### Transwell migration and invasion assays

We conducted a cell migration assay utilizing the Transwell Chamber provided by LABSELECT (#14241). In this assay, cells (0.5-2 × 10^5^) were seeded in the upper chamber with serum-free media. The cell invasion assay followed a similar procedure as the migration assay. Matrigel (#356234, Corning) was applied onto the upper chamber prior to seeding the cells. The lower chamber contained media supplemented with 10% FBS as a chemoattractant. Following an incubation period of 24 to 48 hours, the cells that invaded through the membrane were fixed using a solution of 4% paraf-ormaldehyde for a duration of 15 minutes at room temperature. Subsequently, they were stained with a solution of crystal violet (0.5%) and quantified using ImageJ software.

### RNA immunoprecipitation (RIP) assay

The EZ-Magna RIP RNA-Binding Protein Immunoprecipitation Kit (#17-700, Millipore) was utilized for the RIP assay. First, protein A/G magnetic beads were washed twice with RIP Wash Buffer, resuspended, and incubated with anti-YBX1/IGF2BP1/YTHDC1 or anti-IgG at 4 °C for 30 min. Cells were cultured, washed with PBS, lysed with RIP Lysis Buffer, and the lysate was mixed with the bead-antibody complex and incubated overnight at 4 °C. After incubation, the beads were collected on a magnetic frame, washed four times with RIP Wash Buffer, eluted with RIP Elution Buffer at room temperature for 15 min, and the eluate was transferred. RNA was extracted, dissolved, and its concentration and purity measured. Reverse transcription was performed, and the enrichment of target RNA (LINC02418/CTNNB1) was detected by qPCR.

### MeRIP assay

The Magna MeRIP m6A Kit (#17-10499) was applied for the identification and enrichment of N6-methyladenosine (m6A) modified RNA. The process begins with the extraction of total RNA from CRC cells and the RNA is then fragmented into smaller pieces by either heating or enzymatic digestion, which allows for more efficient binding with the m6A antibody. After fragmentation, the RNA is incubated with the m6A-specific antibody provided in the kit, which selectively binds to the m6A-modified RNA. The antibody-RNA complex is then captured using magnetic beads. Following immunoprecipitation, the bound RNA is washed to remove non-specific binding and then eluted. The m6A-enriched LINC02418 is purified, quantified, and analyzed using qPCR.

### RNA stability assay

Briefly, colorectal cancer (CRC) cells were seeded in 12-well plates for approximately 16-24 hours prior to transfection with siRNA or plasmid. Following a 48-hour transfection period, CRC cells were treated with 5 μg/ml actinomycinD (Abmole, USA) and RNA samples were collected at specified time points. The RNA expression levels were assessed using RT-qPCR and normalized to GAPDH.

### RNA pull-down assay

An RNA pull-down assay was performed using a Pierce™ Magnetic RNA-Protein Pull-Down Kit (#20164, Thermo Scientific) following the manufacturer’s protocol. CRC cells were lysed for 10 minutes and subsequently centrifuged at 13,000×g for 10 minutes at 4 °C to obtain the supernatant including cell lysates, which were then incubated with biotinylated LINC02418 and antisense-LINC02418 RNA probes (Sangon Biotech, Shanghai, China) overnight with streptavidin magnetic beads. The magnetic beads were boiled (10 minutes at 100 °C) to elute proteins from the Protein-RNA complexes, followed by measurement of protein expression using Western blot analysis.

### Luciferase reporter assay

The transcriptional impact was evaluated using a dual-luciferase analysis. The pPro-RB-Report vector (RiboBio, China) was utilized to clone either the WT or MT promoters. These luciferase vectors were co-transfected into CRC cells and incubated for 48 hours. Subsequently, the Dual-Luciferase® Assay Kit (#E1910, Promega) was employed to measure luciferase activity. The activation level of the reporter gene was analyzed by calculating the ratio between the relative luciferase units of firefly luciferase and Renilla luciferase.

### Chromatin immunoprecipitation (ChIP) assay

A ChIP kit (#P2078, Beyotime) was employed to perform the ChIP assay. Cells were cross-linked with 1% formaldehyde for 5 minutes at 37 °C and then the cross-linking was halted by 0.125 M glycine for 10 minutes at room temperature. The cells were rinsed twice using cold PBS supplemented with PMSF (#ST506, Beyotime) and collected in SDS Lysis buffer from the ChIP Assay Kit. Subsequently, the Bioruptor Plus was utilized to sonicate the sample at 4 °C. Protein A + G Agarose/Salmon Sperm DNA was used to pre-clear the whole cell lysate for 30 minutes at 4 °C. After extracting the 2% input sample, the remaining sample was evenly divided and incubated with anti-YBX1 or control IgG antibody overnight. Next, Protein A + G Agarose/Salmon Sperm DNA was added and incubated for 2 hours at 4 °C. Thereafter, the beads were successively washed with Low-Salt Immune Complex Wash Buffer, High-Salt Immune Complex Wash Buffer, LiCl Immune Complex Wash Buffer and TE Buffer (twice) for 5 minutes at 4 °C with rotation. DNA-protein complexes were eluted with elution buffer (1% SDS and 0.1 M NaHCO3) and de-crosslinked by adding 0.2 M NaCl and heating for 4 hours at 65 °C. Then, the proteins were digested with proteinase K for 1 hour at 45 °C, and the DNA fragments were purified by a DNA Purification Kit (#D0033; Beyotime) and applied for qPCR reaction. The ChIP-qPCR primers are available in Supplemental Table 5.

### Nuclear and cytosol fractionation

Nuclear and cytosol fractions were obtained with the application of a Nuclear and Cytoplasmic Protein Extraction Kit (#P0028, Beyotime) in accordance with the pre-existing protocols. Under the condition of low osmotic pressure, the cells experience sufficient expansion. Subsequently, the cell membrane was disrupted, leading to the release of cytoplasmic proteins. The nucleus was then separated by means of centrifugation. Eventually, nuclear protein was extracted using high-salt nuclear protein extraction reagent. PMSF (#ST506, Beyotime) was utilized to prevent protein degradation.

### Animal experiments

Male BALB/c nude mice (4 weeks old) were obtained from Vital River (Beijing, China). The mice received a standard diet for rodents and were kept in a controlled environment with no pathogens present. The room temperature was set at 23 °C with a balanced light-dark cycle of twelve hours each. Following seven days of acclimatization, we randomly assigned the nude mice into two groups named shNC and shLINC02418-1. To establish xenografts through subcutaneous implantation, we injected either 5 × 10^6^ diluted shNC cells or shLINC02418#1 cells mixed with PBS solution (100 μL) into the skin layer of nude mice. Tumor dimensions, including length (a) and width (b), were recorded using a vernier caliper at three-day intervals. The formula: V = 1/2 × a × b^2^ was used to calculate the tumor volume. Subsequently, the mice were sacrificed for weighing and subsequent IHC staining of tumor tissues. For the lung metastasis model, CRC cells (2 × 10^6^ cells in 100 μL PBS per mouse) were injected via the tail vein. After 45 days, their lung tissues were dissected for observation through H&E staining, followed by counting of metastatic nodules.

### Statistical analyses

All statistical analyses were performed using SPSS 23.0 (Chicago, IL, USA) and GraphPad Prism 9.0 (La Jolla, CA, USA). Differences between groups were determined through the utilization of Student’s t-test or one-way ANOVA. Spearman’s correlation coefficient was employed to analyze the associations between different genes. For progression-free survival (PFS), Kaplan-Meier curves were drawn and data were analyzed using log-rank test. *P* < 0.05 was deemed to be statistically significant (*P < 0.05, **P < 0.01, ***P < 0.001). To ensure robustness and reliability of the findings, data from at least three independent replicate experiments were utilized for all statistical analyses performed in this study.

## Supplementary information

Supplementary table and figure

WB.

## Data Availability

The data which was used and/or analyzed during this study is available from the corresponding author on reasonable request.
