# Supplementary material for: m6A-modified LINC02418 induces transcriptional and post-transcriptional modification of CTNNB1 via interacting with YBX1 and IGF2BP1 in colorectal cancer
Source: Cell Death Discov. 2025 Mar 13;11:101. doi: 10.1038/s41420-025-02365-4 (PMC11906587; doi:10.1038/s41420-025-02365-4)
Supplement: Supplementary file 1 — Supplementary table and figure [file 41420_2025_2365_MOESM1_ESM.docx]

**Supplementary Table 1** Sequences of shRNA for LINC02418.

| **shRNA** | **Sequence 5’---3’** |
| --- | --- |
| sh1 | GCGUGAACAUGCCGGGCAATT |
| sh2 | CCGCAUGACCCAUCUGAUUTT |

**Supplementary Table 2** Sequences of siRNA for METTL3, YBX1 and IGF2BP1.

|  | **Sequence 5’---3’** |
| --- | --- |
| **siMETTL3-1** | AGGCAGCTCATCTGTGTCCT |
| **siMETTL3-2** | GGUUGGUGUCAAAGGAAAUTT |
| **siMETTL14-1** | GCTGGACTTGGGATGATATTA |
| **siMETTL14-2** | GAACCTGAAATTGGCAATATA |
| **siWTAP-1** | AGAAUUAACUCAUCUCUUGCC |
| **siWTAP-2** | UAGGAAAAACAAGUUGAUCGC |
| **siYBX1-1** | CAGUUCAAGGCAGUAAAUAUGCA |
| **siYBX1-2** | GTTCAATGTAAGGAACGGAT |
| **siIGF2BP1-1** | TTTACTTCCTCCTTGGGACTT |
| **siIGF2BP1-2** | GGCCAGUUCUUGGUCAAAUTT |

**Supplementary Table 3** The primer sequences of qPCR.

| **Gene** | **Forward primer** | **Reverse primer** |
| --- | --- | --- |
| LINC02418 | ATTTCCATGGCGTTTCTCAC | AGGCAGGAGAATTGCTTGAA |
| METTL3 | AGGCAGCTCATCTGTGTCCT | GCTTGGCGTGTGGTCTTT |
| YBX1 | GGGGACAAGAAGGTCATCGC | CGAAGGTACTTCCTGGGGTTA |
| IGF2BP1 | AGTGTGCTGGGAGAAGAGGAAG | TTGCGGTTGTCTTGTTGTTACTGTTG |
| CTNNB1 | AAAGCGGCTGTTAGTCACTGG | CGAGTCATTGCATACTGTCCAT |
| cyclin D1 | GCTGCGAAGTGGAAACCATC | CCTCCTTCTGCACACATTTGAA |
| Axin 2 | TACACTCCTTATTGGGCGATCA | TTGGCTACTCGTAAAGTTTTGGT |
| c-MYC | GTCACACCCTTCTCCCTTCG | CGGGTCGCAGATGAAACTCT |
| GAPDH | GTCTCCTCTGACTTCAACAGCG | ACCACCCTGTTGCTGTAGCCAA |

**Supplementary Table 4** The information of antibodies.

| **Antibodies** | **SOURCE** | **IDENTIFIER** |
| --- | --- | --- |
| YBX1 | Abcam | Ab76149 |
| IGF2BP1 | Abcam | Ab100999 |
| c-MYC | Proteintech | 10828-1-AP |
| β-catenin | Cell Signaling Technology | 9562 |
| cyclin D1 | Cell Signaling Technology | 2922 |
| Axin 2 | Abcam | Ab185821 |
| Ki-67 | Abcam | Ab15580 |
| EMT Antibody Sampler Kit | Cell Signaling Technology | 9782 |
| Tubulin | ZSGB-BIO | TA-10 |
| Lamin B1 | Cell Signaling Technology | D9V6H |
| β-actin | ZSGB-BIO | TA-09 |
| GAPDH | Thermo Scientific | 39-8600 |

**Supplementary Table 5** The primer sequences of CTNNB1 and LINC02418 promoter used in the ChIP assay.

| **Promoter** | **Forward primer** | **Reverse primer** |
| --- | --- | --- |
| CTNNB1 promoter | CTCAGACGGCAGCAGACT | CTGTATCCTGCCGCCACC |
| LINC02418 promoter | CAAGACACAACTGTAGGCCA | AGTCCTCTGCCTGTTTACTGT |


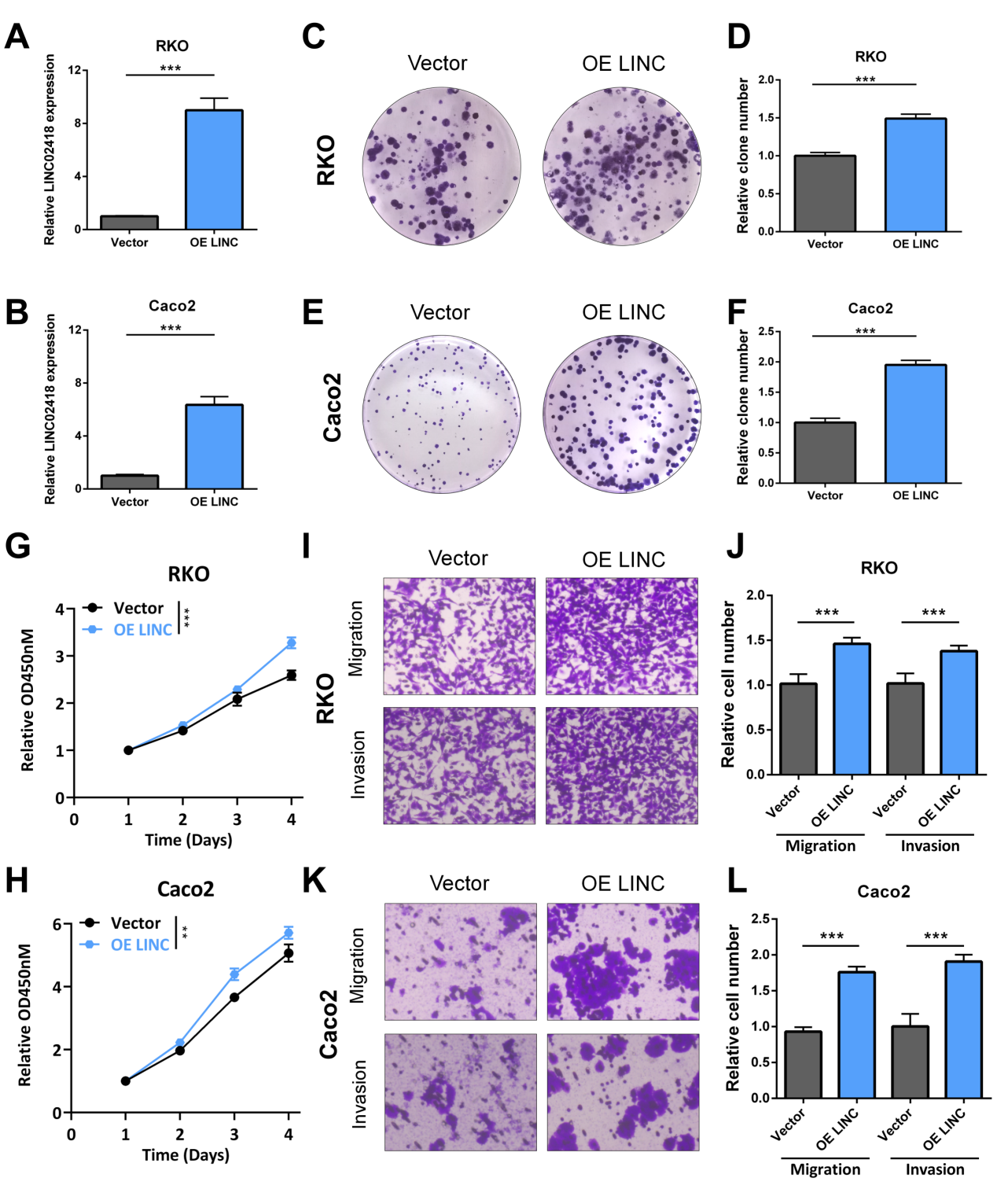


**Supplementary Figure 1** LINC02418 overexpression promotes proliferation and metastasis of colorectal cancer cells in vitro. (A-B) The expression of LINC02418 was determined by qPCR in RKO and Caco2 cells with LINC02418 overexpression. (C-D) Representative images and quantification of colony formation assay depicting the growth of RKO cells under LINC02418 overexpression. (E-F) Representative images and quantification of colony formation assay depicting the growth of Caco2 cells under LINC02418 overexpression. (G-H) CCK8 assay indicates the change in cell viability of RKO and Caco2 cells under LINC0248 overexpression. (I-J) Representative images and quantification of transwell assay depicting the migration and invasion abilities of RKO cell with LINC02418 overexpression. (K-L) Representative images and quantification of transwell assay depicting the migration and invasion abilities of Caco2 cell with LINC02418 overexpression.


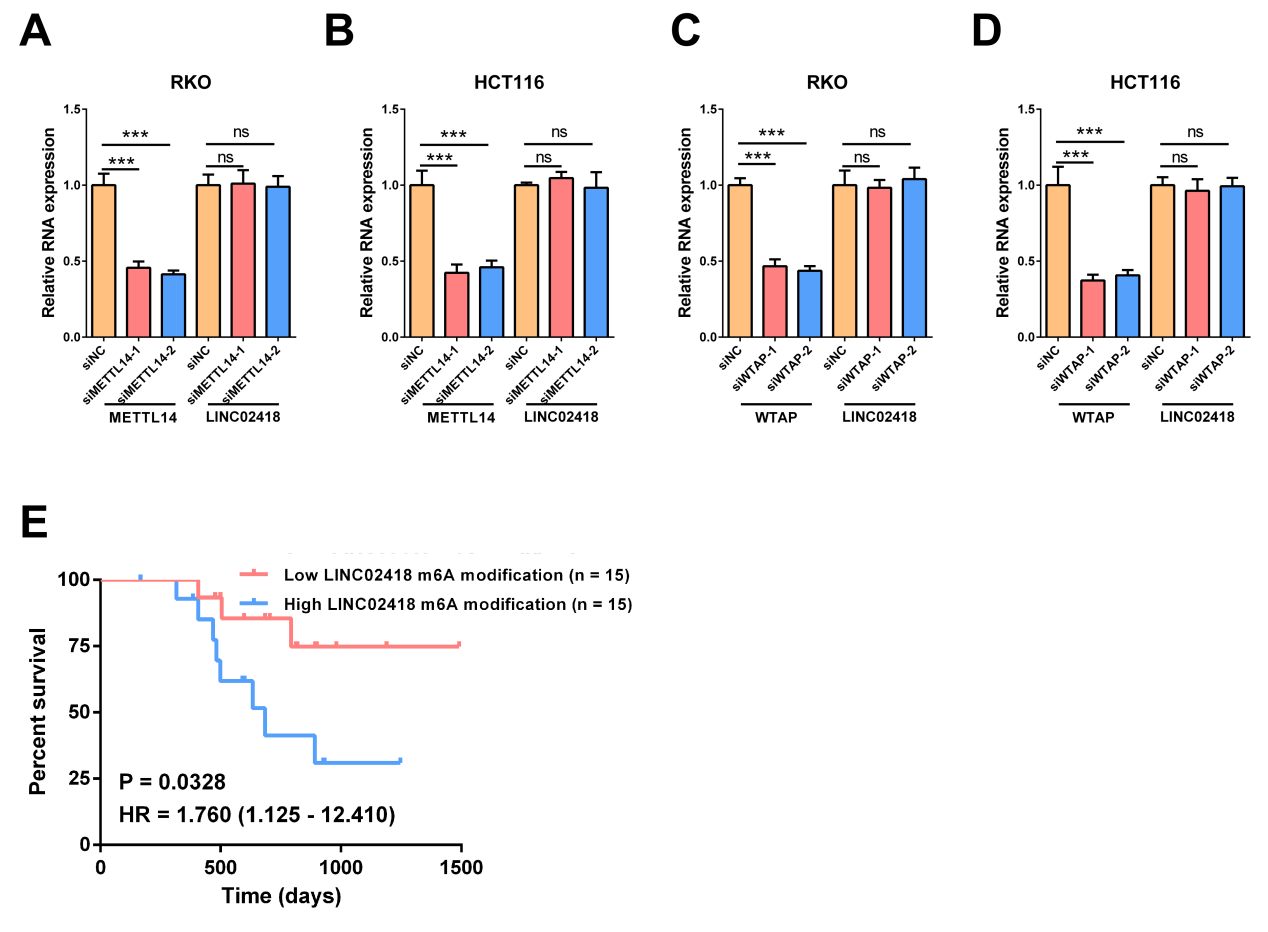


**Supplementary Figure 2** (A-B) LINC02418 expression was detected by qPCR after METTL14 knockdown in RKO and HCT116 cells. (C-D) LINC02418 expression was detected by qPCR after WTAP knockdown in RKO and HCT116 cells. (E) High LINC02418 m6A modification level was associated with worse overall-survival in CRC patients.

**
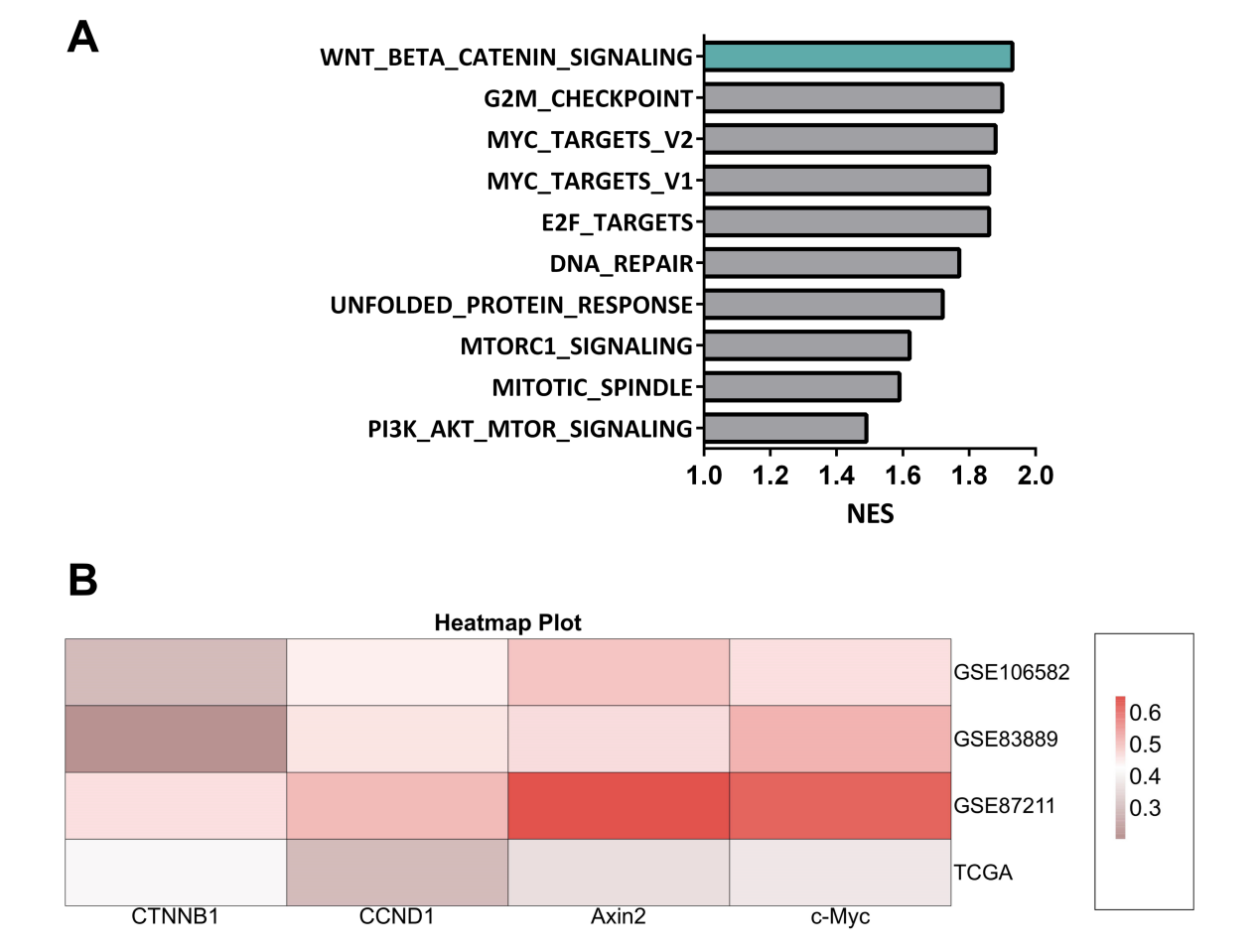
**

**Supplementary Figure 3** (A) The enrichment scores of LINC02418 with different pathways in the GSEA analysis. (B) Correlation of gene expression between LINC02418 and β-catenin and typical Wnt signaling pathway-targeted genes in GEO (GSE106582, GSE83889 and GSE87211) and TCGA databases.

**
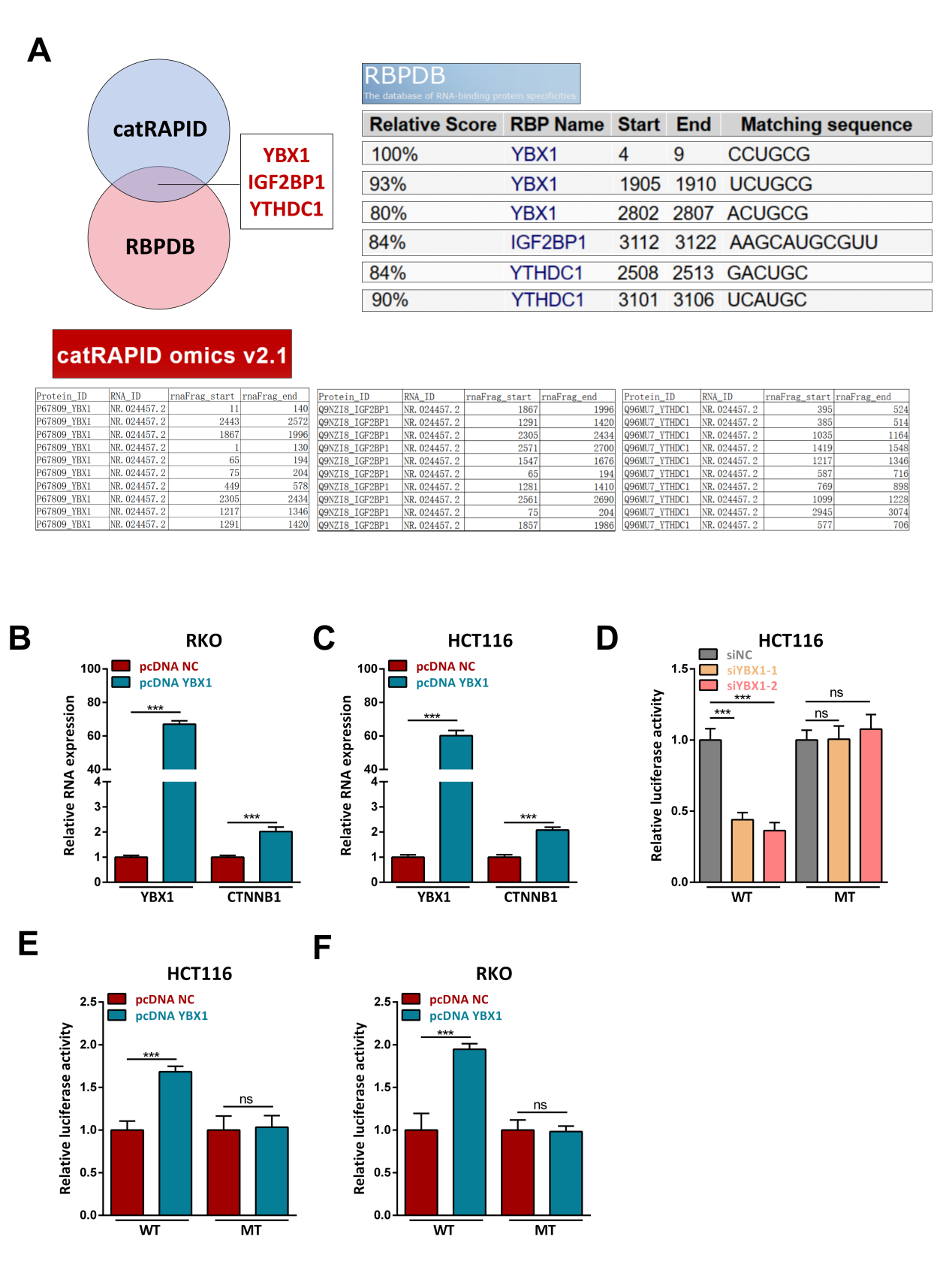
**

**Supplementary Figure 4** (A) The Venn diagram for carRAPID and RBPDB databases was used to reveal that LINC02418 might interact with YBX1, IGF2BP1 and YTHDC1. (B-C) β-catenin expression was detected by q-PCR after YBX1 overexpression in RKO and HCT116 cells. (D) The dual-luciferase analysis for β-catenin transcriptional activity was performed in HCT116 cell with or without YBX1 knockdown. (E) The dual-luciferase analysis for β-catenin transcriptional activity was performed in HCT116 cell with or without YBX1 overexpression. (F) The dual-luciferase analysis for β-catenin transcriptional activity was performed in RKO cell with or without YBX1 overexpression.

**
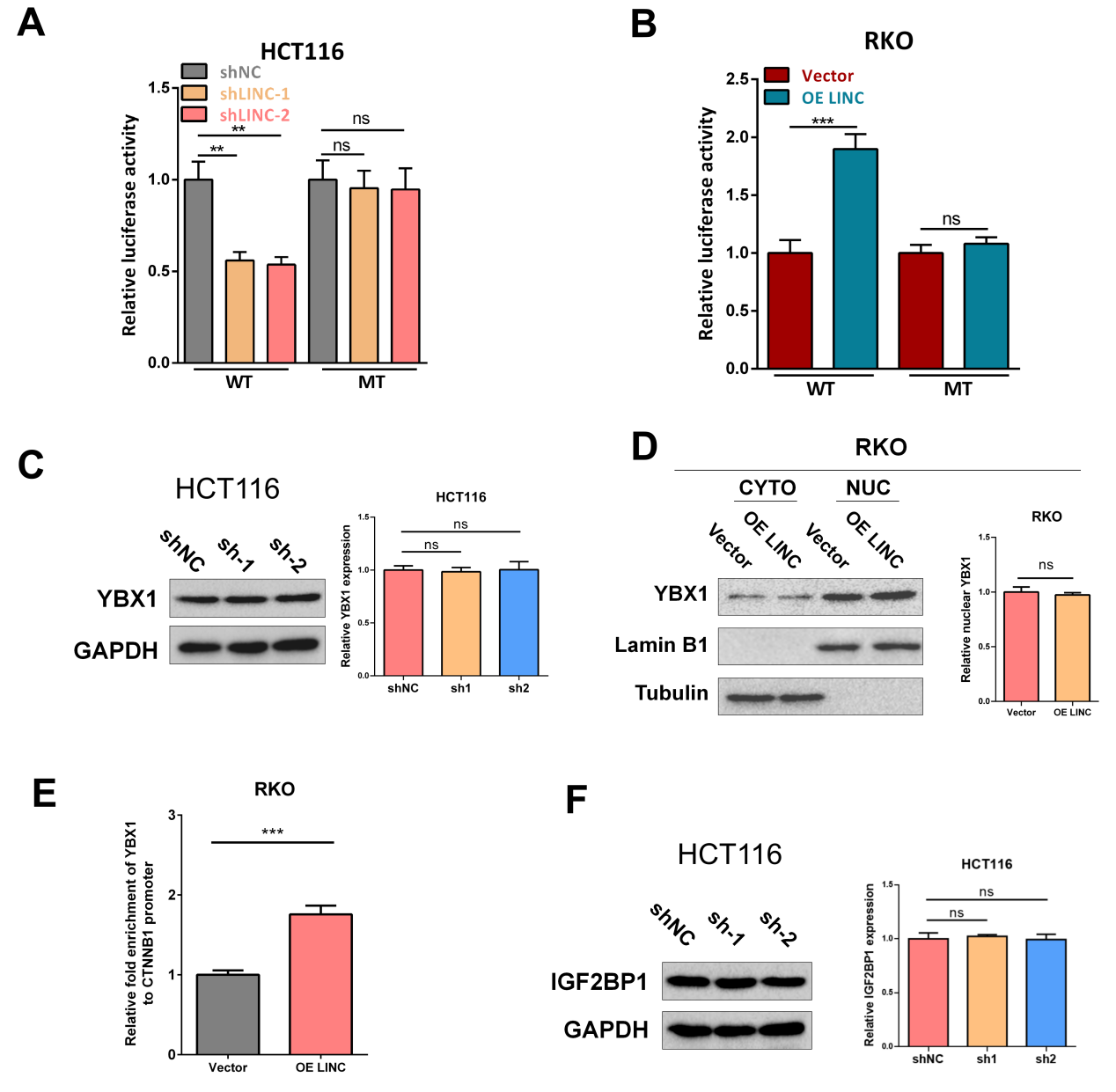
**

**Supplementary Figure 5** (A)The dual-luciferase analysis for β-catenin transcriptional activity was performed in HCT116 cells with or without LINC02418 knockdown. (B) The dual-luciferase analysis for β-catenin transcriptional activity was performed in RKO cell with or without LINC02418 overexpression. (C) YBX1 expression was detected by western blotting after in HCT116 cell with LINC02418 knockdown. (D) Western blot analysis to show the YBX1 expression in the nucleus and cytoplasm of RKO cell with LINC02418 overexpression. (E) CHIP-qPCR showed the binding ability of YBX1 to the CTNNB1 promoter could be induced in RKO cell with LINC02418 overexpression. (F) IGF2BP1 expression was detected by western blotting after in HCT116 cell with LINC02418 knockdown.

**
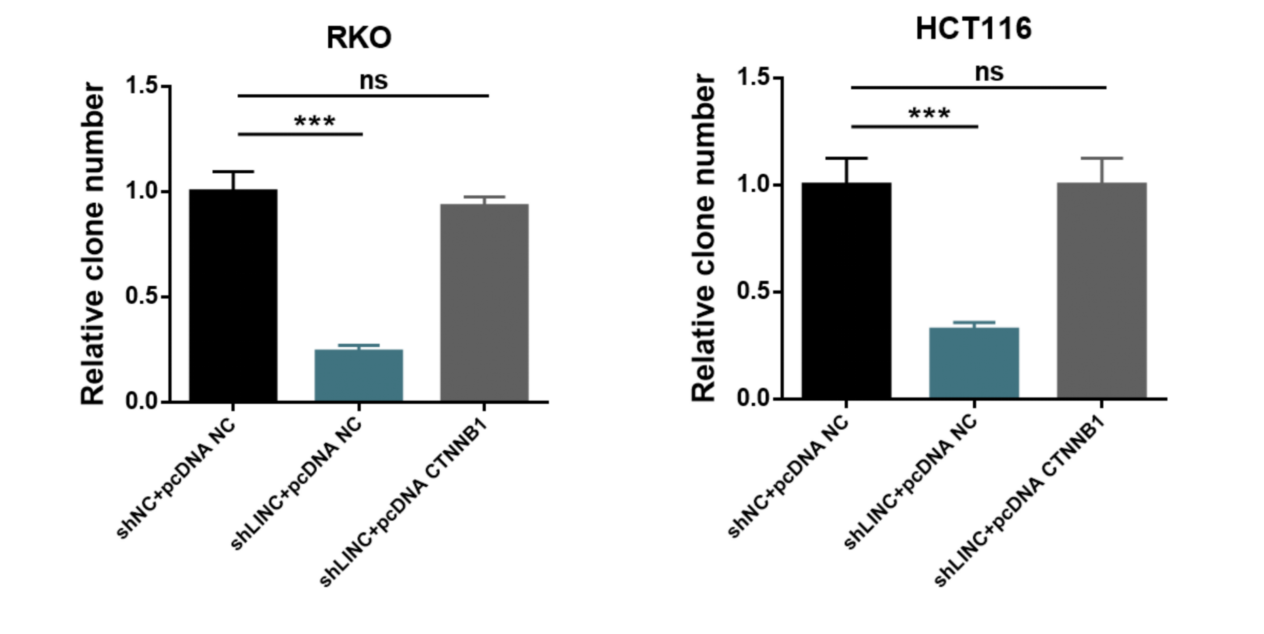
**

**Supplementary Figure 6** Quantification of colony formation assay in the rescue experiment.

**
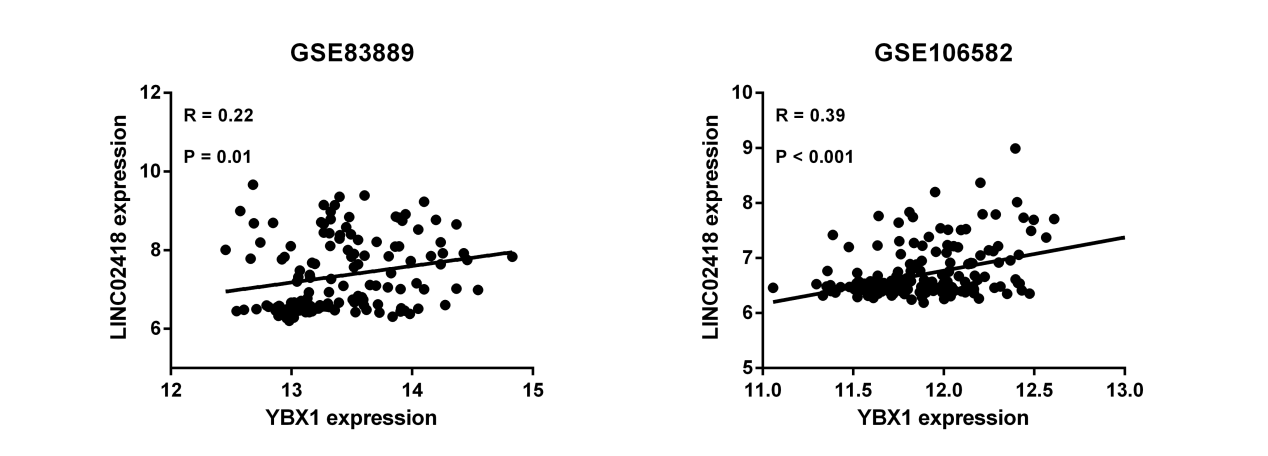
**

**Supplementary Figure 7** The correlation between the LINC02418 and YBX1 expression levels in the GSE83889 and GSE106582 databases.
