# Supplementary figures and images for: m6A-modified LINC02418 induces transcriptional and post-transcriptional modification of CTNNB1 via interacting with YBX1 and IGF2BP1 in colorectal cancer
Source: Cell Death Discov. 2025 Mar 13;11:101. doi: 10.1038/s41420-025-02365-4 (PMC11906587; doi:10.1038/s41420-025-02365-4)

Fig 4F-G


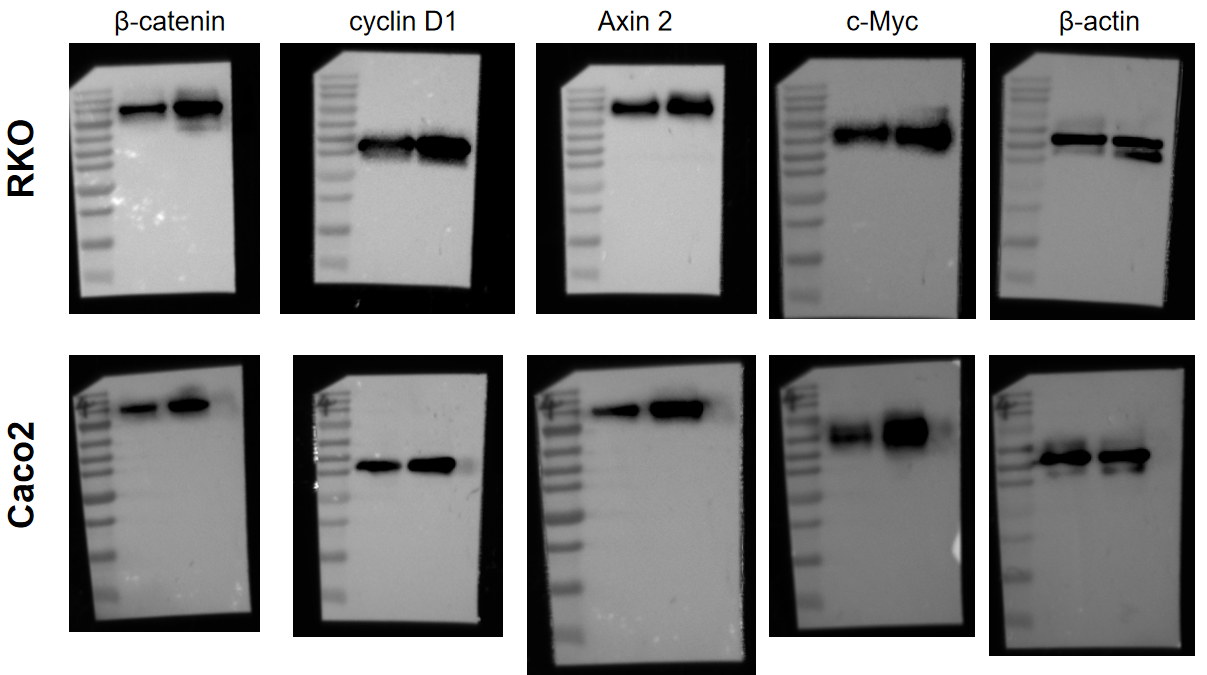


Fig 4H-I


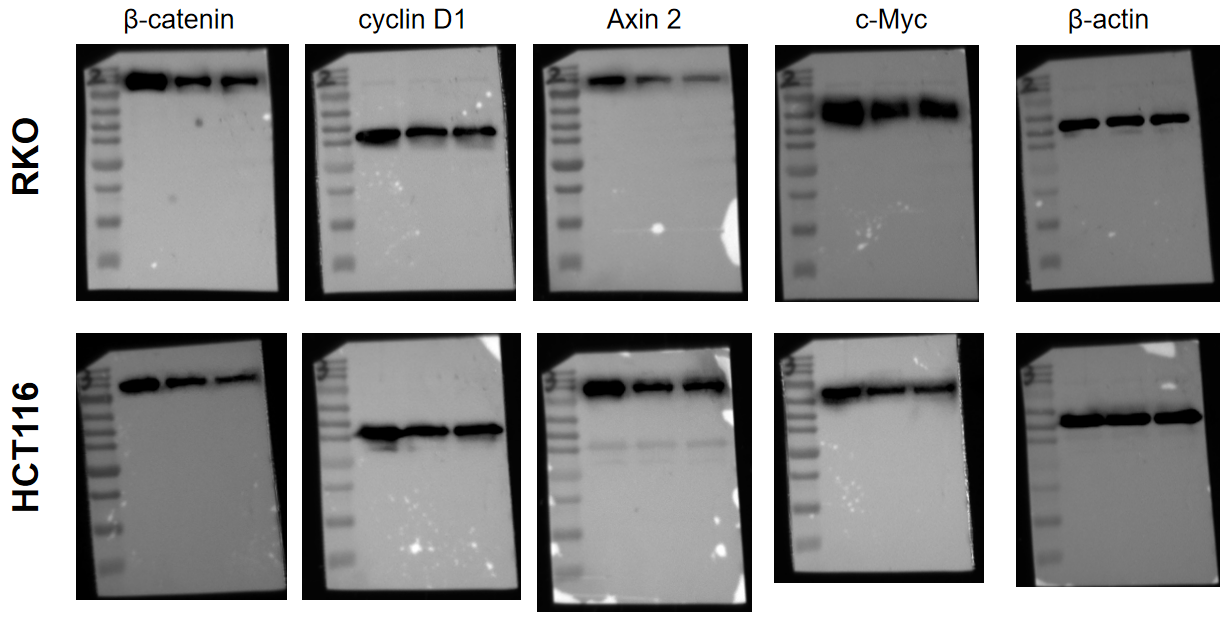


Fig 5D


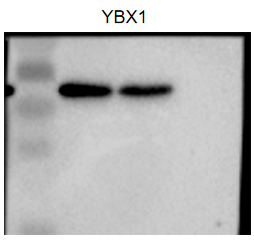


Fig 5N


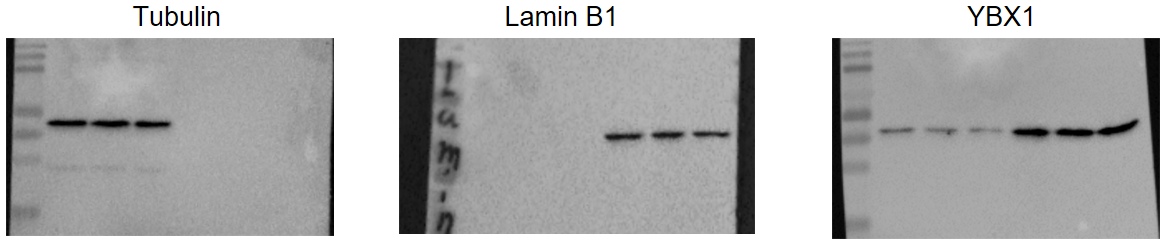


Fig 6A


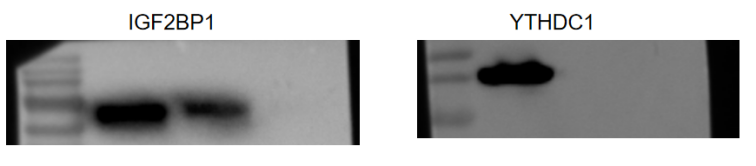


Fig 7I


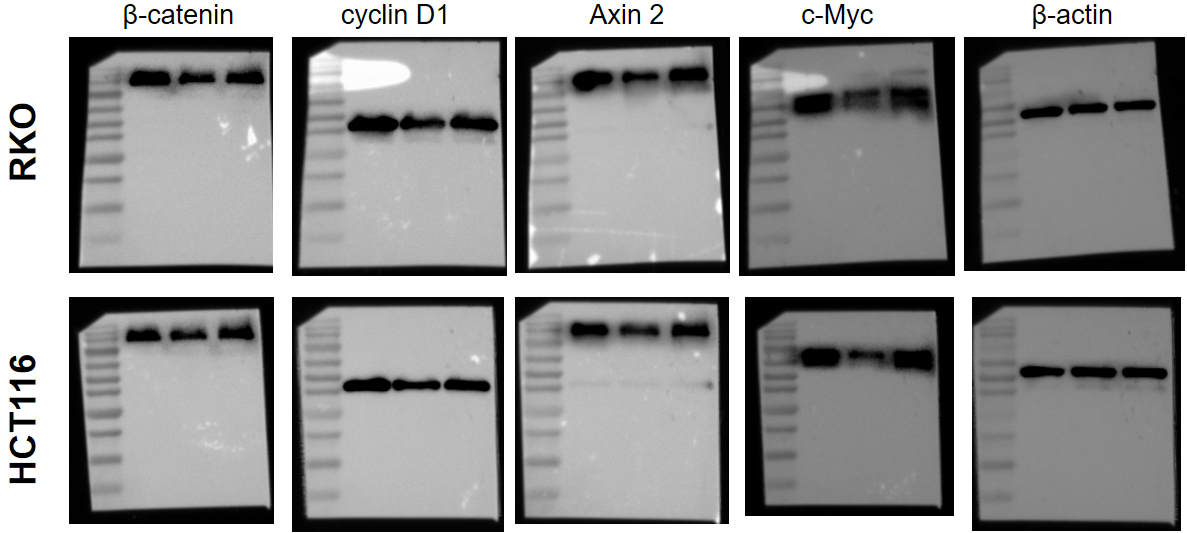


Fig S4C


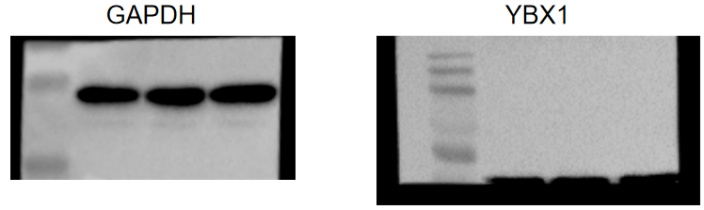


Fig S4D


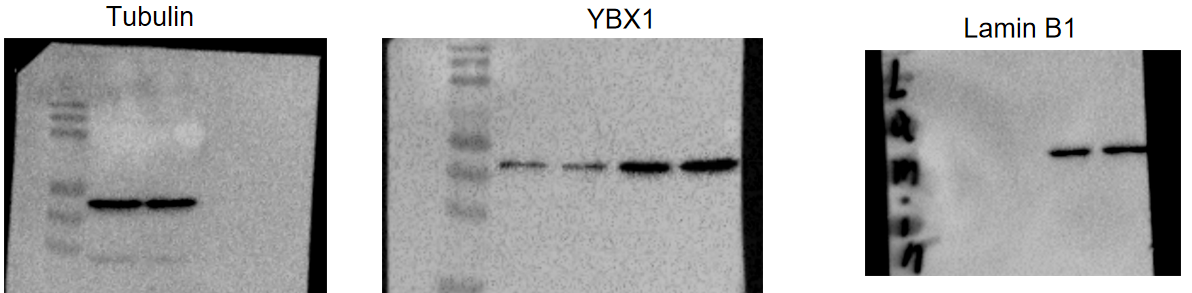

Supplement: Supplementary file 2 — WB. [file 41420_2025_2365_MOESM2_ESM.docx]
